# Nutrient intake and its possible drivers in free‐ranging European brown bears (*Ursus arctos arctos*)

**DOI:** 10.1002/ece3.10156

**Published:** 2023-05-30

**Authors:** Annelies De Cuyper, Diederik Strubbe, Marcus Clauss, Luc Lens, Andreas Zedrosser, Sam Steyaert, Leen Verbist, Geert P. J. Janssens

**Affiliations:** ^1^ Department of Veterinary and Biosciences, Faculty of Veterinary Medicine Ghent University Merelbeke Belgium; ^2^ Terrestrial Ecology Unit, Department of Biology, Faculty of Sciences Ghent University Ghent Belgium; ^3^ Clinic for Zoo Animals, Exotic Pets and Wildlife, Vetsuisse Faculty University of Zurich Zurich Switzerland; ^4^ Department of Natural Sciences and Environmental Health, Faculty of Technology, Natural Sciences and Maritime Sciences University of South‐Eastern Norway Bø Norway; ^5^ Institute for Wildlife Biology and Game Management University for Natural Resources and Life Sciences Vienna Austria; ^6^ Faculty of Biosciences and Aquaculture Nord University Steinkjer Norway; ^7^ Onderzoekskern Salto, Odisee Hogeschool, Campus Sint‐Niklaas Sint‐Niklaas Belgium

**Keywords:** Brown bear, metabolism, nutrient ratios

## Abstract

The dietary nutrient profile has metabolic significance and possibly contributes to species' foraging behavior. The brown bear (*Ursus arctos*) was used as a model species for which dietary ingredient and nutrient concentrations as well as nutrient ratios were determined annually, seasonally and per reproductive class. Brown bears had a vertebrate‐ and ant‐dominated diet in spring and early summer and a berry‐dominated diet in fall, which translated into protein‐rich and carbohydrate‐rich diets, respectively. Fiber concentrations appeared constant over time and averaged at 25% of dry matter intake. Dietary ingredient proportions differed between reproductive classes; however, these differences did not translate into a difference in dietary nutrient concentrations, suggesting that bears manage to maintain similar nutrient profiles with selection of different ingredients. In terms of nutrient ratios, the dietary protein to non‐protein ratio, considered optimal at around 0.2 (on metabolizable energy basis), averaged around 0.2 in this study in fall and around 0.8 in spring and summer. We introduced the minimal non‐fat to fat ratio necessary for efficient maintenance metabolism. This ratio varied across seasons but never fell beneath the theoretically estimated minimum to ensure metabolic efficiency. This population thus managed to ingest diets that never exerted a lack of glucogenic substrate, suggesting that metabolic efficiency may either be a driver of active diet selection or that natural resources available to bears did not constitute a constraint in this respect. Given the considerable proportion of fiber in the diet of brown bears, the relevance of this nutrient and its role in foraging behavior might be underestimated.

## Graphical abstract

Dietary nutrient concentrations (incl. fiber) and ratios of free‐ranging Swedish brown bears showed that: the diet is protein‐dominated in spring and early summer, carbohydrate‐dominated in fall and constant in fiber throughout seasons; bears managed to obtain similar dietary nutrient profiles with different ingredients; the protein to non‐protein ratio only averaged around 0.2 in fall and the fat to non‐fat ratio varied across seasons but never fell beneath the theoretically estimated minimum to ensure metabolic efficiency.

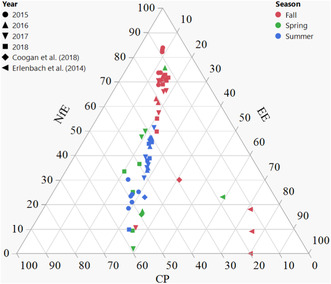

## 1 INTRODUCTION

Diet composition, i.e. the dietary ingredients that make up the food regime of an organism, have been studied across a suite of free‐ranging species (e.g. Rogers et al., 2004, Hayward et al., 2006, Bravo et al., 2017). Foraging behavior has traditionally been explained in light of the optimal foraging theory (OFT), where it is assumed that animals strive to maximize their fitness by means of maximizing net energy intake (Pyke et al., 1977). Recent evidence suggests that the nutrient composition of the diet is an important factor to consider when studying foraging behavior (Coogan et al., 2017; Rosenblatt & Schmitz, 2016; Rothman et al., 2007). Apart from functioning as an energy source, nutrients (typically protein, fat and carbohydrates) also fulfill specific metabolic functions (McDonald et al., 2011). Increasing evidence shows that the intake of these nutrients in specific ratios has metabolic significance. For example, for several invertebrates and vertebrates, nutrient composition influences the fitness of species, their reproduction and longevity (Jensen et al., 2012; Lee et al., 2008; Solon‐Biet et al., 2014), immune system functioning (Cotter et al., 2011), growth and body mass (Simpson et al., 2004) and fat mass gain in hibernating animals (Erlenbach et al., 2014; Robbins et al., 2007). Therefore, instead of approaching food or diet only in terms of energy, the study of nutrients and their balance in the diets of free‐ranging animals can increase our knowledge on the mechanisms underpinning foraging behavior.

An increasing number of studies reported on the dietary nutrient profiles (protein, carbohydrates and fat) of free ranging species (e.g. Bosch et al., 2015; Gazzola & Balestrieri, 2020; Rothman et al., 2007). Additionally, self‐selection diet studies (i.e. individuals can select from a variety of foods offered ad libitum) with species under human care increased knowledge on the nutrient composition that would be ingested when no restrictions are imposed (e.g. Erlenbach et al., 2014; Felton et al., 2016; Hewson‐Hughes et al., 2013). Furthermore, the nutrients typically considered are mainly restricted to (digestible) protein and carbohydrates, and fat (Bosch et al., 2015; Coogan et al., 2018; Erlenbach et al., 2014), but little attention has been given to fiber (indigestible but fermentable carbohydrates or protein) as a separate main nutrient. Fiber is the main energy source for herbivores (Stevens & Hume, 1998) and fulfills several physiological functions other than just energy provision (Dearing & Kohl, 2017). In herbivores, nutrient optimization is mostly studied as the ratio protein to non‐protein (fat, digestible carbohydrates and fiber), with no specific fiber target included (Felton et al., 2016; Rothman et al., 2011). In carnivores, the importance of fiber remains underestimated in comparison to protein, carbohydrates, and fat. Whole prey, however, contains indigestible proteinaceous fractions such as tendons, bone, fur, connective tissue and hair, that is, animal fiber (Depauw et al., 2012), which is important for gut health (less gut putrefaction and inflammation) and regulates fermentation patterns in the carnivore gut (De Cuyper et al., 2018; Depauw et al., 2012, 2014; Whitehouse‐Tedd et al., 2015) and even affects systemic health (Depauw et al., 2013). Here, we address the nutrient knowledge gap by studying the nutrient profiles of brown bears.

Feeding habits of brown bears (*Ursus arctos*) have been studied elaborately across the globe and diet composition plays an essential role in their reproduction, fitness and longevity (Hilderbrand et al., 1999; Robbins et al., 2012; Zedrosser et al., 2006). Described as omnivorous carnivores, generalist omnivores, opportunistic omnivores, polyphagous or having a catholic diet, brown bears are flexible consumers of diets consisting of a variety of animal‐ and plant‐based foods (Bojarska & Selva, 2012; Juniper, 2007; Stenset et al., 2016). The actual diet composition of bears depends on many factors at an intrinsic level (e.g. age, sex, energetic requirements) (Rode et al., 2006b) as well as an external level (food availability) (Bojarska & Selva, 2012), both subject to spatial and temporal variations (Bojarska & Selva, 2012; Munro et al., 2006; Stenset et al., 2016). Within populations, the seasonal and annual abundance of food resources strongly determines the diet of brown bears (Mattson et al., 1991; McLellan & Hovey, 1995; Munro et al., 2006). Apart from temporal effects, social organization in bear populations can affect resource use. Despotism is a social structure where dominant individuals displace conspecifics from high quality habitats (Fretwell & Lucas, 1969). This can determine habitat use and lead to spatiotemporal segregation in ursids (Elfström, Zedrosser, Støen, & Swenson, 2014) which can ultimately affect resource use (Ben‐David et al., 2004; Blanchard & Knight, 1991; Steyaert, Reusch, et al., 2013). Sexually selected infanticide (i.e., dominant males kill unrelated young to increase the chances of mating) has been documented in the southcentral Swedish brown bear population during the mating season. This phenomenon leads to spatiotemporal avoidance of males by females with cubs‐of‐the‐year (Steyaert, Kindberg, et al., 2013), and ultimately affects the quality (defined as low fecal protein and high fecal lignified fiber) of the latter (Steyaert, Reusch, et al., 2013). Similarly, subadult individuals actively avoid high quality habitats dominated by adult males due to the increased risk of intraspecific predation (Elfström, Zedrosser, Jerina, et al., 2014; Elfström, Zedrosser, Støen, & Swenson, 2014). Diets of dominant conspecifics typically appear to show higher proportions of (vertebrate) prey (Ben‐David et al., 2004; Elfström, Davey, Zedrosser, et al., 2014; Steyaert, Reusch, et al., 2013).

Plenty of information exists on the ingredient composition of ursid diets across regions, years and seasons; however, information of the dietary nutrient composition is less extensive although recently emerging (Coogan et al., 2018; Erlenbach et al., 2014; López‐Alfaro et al., 2015). Generally, spring and summer diets tend to be protein‐dominated due to the heavy reliance on vertebrates and insects in many populations, whereas fall diets are typically high in carbohydrates due to fruit growth, hence fruit intake in this period (Coogan et al., 2018; López‐Alfaro et al., 2015; Stenset et al., 2016). Research in the field of nutritional ecology demonstrated that the intake of nutrients in specific ratios has metabolic effects in bears (Erlenbach et al., 2014; Robbins et al., 2007). Initially, it was noticed that brown bears with access to spawning salmon (*Onchorynchus* spp.) chose to intermix their diet with fruits, which suggests that they did not select for the diet with the highest possible energy return (Fortin et al., 2007; Rode et al., 2006a). Studies with grizzly bears in human care showed that bears self‐selected for a mixed fruit‐salmon diet when both were offered (Robbins et al., 2007). On a nutrient level, captive grizzly bears selected for a diet with 17% protein and 83% non‐protein (carbohydrates and fat) which led to a protein to non‐protein ratio of ~0.2 on a metabolizable energy basis (Erlenbach et al., 2014). This specific ratio was considered an optimum, since it could be linked to maximizing weight gain (fat mass) per energy unit, which is an important metabolic effect for bears preparing for hibernation. The 0.2 ratio in free‐ranging bears can only be obtained whenever sufficient carbohydrate or fat resources are available, which is typically the case in late summer and fall (Coogan et al., 2014). More recently, this comparatively low protein ratio has been suggested to be a common physiological characteristic of ursids, and higher protein provision has been linked to disease in zoo‐kept bears of a variety of species (Robbins, Christian, et al., 2022; Robbins, Tollefson, et al., 2022; Rode et al., 2021). However, although Coogan et al. (2018) generally observed that free‐ranging brown bears (worldwide) are close to this optimum in fall during the hyperphagic period, this was more the case for bears relying on anthropogenic foods and less when relying on natural foods. The latter showed more variable protein to non‐protein ratios, often with higher dietary protein concentrations, suggesting that the realized nutrient ratio depends on the specific conditions of each bear population. Furthermore, López‐Alfaro et al. (2013) highlighted the importance of high protein diets in early spring to replenish the protein losses from early lactation and to maintain high protein concentrations throughout lactation in female bears. It therefore seems that apart from the fat storage in fall, other factors may determine the nutritive value of the bear's diet throughout the year.

Building on an extensive dataset from Swedish brown bears, tracked by the Scandinavian Brown Bear Research Project in southcentral Sweden from 2015 to 2018, we determined ingredient and nutrient (including fiber) levels in the diet of this population, and evaluated nutrient ratios (including the protein to non‐protein ratio [Erlenbach et al., 2014] and other combinations) on a seasonal and annual basis as well as for every reproductive class. We expected vertebrate‐ and ant‐dominated diets in spring and early summer and a berry‐dominated fall diet (Stenset et al., 2016; Swenson et al., 1999). Given this ingredient composition, we expected high dietary protein intakes in spring and summer with a gradual decline toward fall and an inverse development for carbohydrates. We additionally aimed at describing fiber concentrations (plant and animal fiber) in the diet of free‐ranging brown bears as a basis for further investigating its role in carnivore digestive physiology. Given the previously described differences in diet selection between reproductive classes, we expected a higher proportion of (vertebrate) prey; hence a higher protein content in the diets of adult males and lone females versus females with offspring during the mating season, and subadult bears, respectively (Ben‐David et al., 2004; Elfström, Davey, Zedrosser, et al., 2014; Steyaert, Reusch, et al., 2013). In terms of nutrient ratios, we expected to find a protein to non‐protein ratio around 0.2 (metabolizable energy basis) in fall as suggested by Erlenbach et al. (2014), however not in spring and summer. Finally, we explored other nutrient ratios (non‐fat to fat ratio) than the protein to non‐protein ratio which might hold meaningful insights into the metabolic significance of nutrient selection.

## 2 MATERIALS AND METHODS

### 2.1 Study area

Brown bears were studied in Dalarna and Gävleborg counties (ca. 61°5′ N, 15°05′ E) in southcentral Sweden, 2015–2018. The area can be characterized as rolling terrain with altitudes ranging from 250 to 650 m above sea level (Hertel et al., 2016). Forests are coniferous and dominated by Scots pine (*Pinus sylvestris*) and Norway spruce (*Picea abies*; Elfström et al., 2008; Swenson et al., 1999) and are intensively managed with ca. 40% of trees being less than 35 years old (Swenson et al., 1999). Mean temperatures range from −7°C in January to 15°C in June, snow cover lasts from late October to early May, and mean precipitation ranges between 350 and 450 mm during the growing season (Frank et al., 2015; Swenson et al., 1999). Brown bear density in the study area (ca. 12,000 km^2^) was estimated at 15–30 bears per 1000 km^2^ (Rivrud et al., 2019).

### 2.2 Brown bear monitoring and scat collection

Every year, the Scandinavian Brown Bear Research Project (SBBRP) tags 40–50 brown bears with GPS‐GSM collars (Vectronic Aerospace GmBh, Berlin, Germany). The procedures required for capturing and handling brown bears (described in Arnemo & Evans, 2017) are approved by the Animal Ethical Committee in Uppsala, Sweden (*C18/15*) and the Swedish Environmental Protection Agency (*NV‐00741‐18*). The GPS collars are programmed to record at least one GPS location per hour. Part of the SBBRP monitoring program focusses on documenting diet composition through scat analysis. For this reason, scats from tracked bears were collected throughout the active period (April/May until late October) in a frequency of 1 scat/individual/week (up until 2015) or 1 scat/individual/2 weeks (from 2016 on). Brown bears commonly defecate close to their resting sites or ‘beds’ (Steyaert et al., 2012). Bear beds were defined as indentations of ca. 1–2 m^2^ in the soil where presence of bears could be confirmed (hair or scat; Steyaert et al., 2019) and located where GPS locations clustered (minimum three consecutive locations within a radius of 30 m for more than 1.5 h; Ordiz et al., 2011; Rauset et al., 2012). Scats were collected within a 5 m radius around a bed. In the mating season, scats were only sampled in locations where only one bed was present, to avoid collecting scats from other individuals than the radio‐collared bear. In the case of females with cubs, only the mother scat was sampled (differentiation via scat size). All scats were collected in plastic bags and stored the same day as collection at −20°C until further analysis.

### 2.3 Scat analyses for dietary components/ingredients

Every scat was weighed, and scat volume was determined by water displacement. Dietary ingredients were determined following the methods of Hamer and Herrero (1987), Dahle et al. (1998) and Stenset et al. (2016). Each scat was homogenized and then thoroughly washed over a 0.6 mm sieve. Thereafter, five subsamples with a volume of 5 mL were taken from the scat. Every subsample was visually examined for dietary ingredients to the most detailed taxonomic level possible. Ants were additionally examined with a 6.3–30 stereoscope and 40–630 microscope. The volume percentage of every ingredient was assessed visually in every subsample which corresponds well with exact volume determination (Mattson et al., 1991) after which the average of the five subsamples was calculated. Diet ingredient categories were: berries (bilberry *Vaccinium myrtillus*, lingonberry *V. vitis‐idaea*, crowberry *Empetrum nigrum*, raspberry *Rubus idaeus*, other berries [unidentified]); other fruit (unidentified); invertebrates (ants [*Formica* spp., *Camponutus* spp. and other ants {unidentified}], other invertebrates [unidentified]); vertebrates (moose *Alces alces*, other vertebrate species, bear hair); vegetation (graminoids, oats *Avena sativa*, horsetail *Equisetum arvense*, maize *Zea mays*, mushrooms [unidentified], leaves and twigs, bilberry bushes, grains/cereals [unidentified], other vegetation [unidentified]); miscellaneous (material that could not be identified or attributed to any of the aforementioned food item categories); bird eggs.

### 2.4 Calculation of the dietary nutrient composition

The percent volume of diet ingredients was used to calculate the diet and nutrient composition of the diet consumed per scat as follows:

- Calculation of the estimated dietary content (EDC): diet ingredients have a different digestibility; hence the proportion of easily digestible diet ingredients may be underestimated in the feces in comparison to the actual biomass ingested. Establishing diet ingredient composition solely based on percent volume (or fecal volume [Vf]) in the scats is therefore biased (Hewitt & Robbins, 1996). Correction factors (CF) were used to take into account the digestibility of diet ingredients and to estimate the actual proportion of diet ingredients in the diet (Bojarska & Selva, 2013; Hewitt & Robbins, 1996; see also online Dryad repository) following the formula:
$${\mathrm{EDC}}_{i}=\left({\mathrm{Vf}}_{i}\times {\mathrm{CF}}_{i}\right)÷\sum \left(\mathrm{Vf}\times \mathrm{CF}\right)$$
  with EDC expressed as % dry matter (DM) of the *i* ^th^ diet ingredient ingested and CF_i_ equal to the dry matter of diet ingredient *i* ingested (g)/fecal volume of the residue of diet ingredient *i* (mL). Scats with more than 5% EDC miscellaneous were omitted from the dataset to avoid any leveraging effect because no nutrient composition can be attributed to this category (López‐Alfaro et al., 2015).
- Nutrient intake profiles: the nutrient composition of every diet ingredient was extracted from the literature (online Dryad repository), i.e. DM, crude protein (CP), crude fat (ether extract, EE), ash, fiber as crude fiber (CF) and total dietary fiber (TDF) (as % DM). Digestible carbohydrates (NfE) were calculated following the formula:
100−CP−EE−Ash−TDF
  Priority was given to subtracting TDF instead of CF given the fact that it is a more complete measure for fiber. For vertebrate diet items (i.e. whole prey), TDF values were extracted from Pritchard and Robbins (1990), where the method was adjusted in order to capture protein‐rich animal fiber. Digestible carbohydrate (NfE) values for whole prey were calculated as 100‐CP‐EE‐ash, without TDF subtraction, given the fact that animal matter TDF consists solely of proteinaceous material, hence is already captured in the CP fraction (see also Discussion). Similarly, for digestible carbohydrate determination of invertebrates, the formula 100‐CP‐EE‐ash was used given the fact that a considerable part of the fiber fraction consists N‐rich chitin, which is captured in the CP fraction (Finke, 2007). The metabolizable energy (ME) of the diet ingredients was calculated with unmodified Atwater factors (16.72 kJ/g protein; 37.62 kJ/g fat; 16.72 kJ/g NfE).
  The nutrient composition of the diet was calculated with the formula:
$$\text{Nutrient concentration diet}\;\left(\%\mathrm{DM}\right)=\sum {\mathrm{EDC}}_{\text{ingredient}}\times {\text{nutrient concentration}}_{\text{ingredient}}$$
- The nutrient composition of the diet as ingested was used to calculate the protein to non‐protein ratio CP:(EE + NfE) as well as the non‐fat to fat ratio (CP + NfE):EE both on DM and ME basis.

### 2.5 Data compilation

The ingredient and nutrient composition of Swedish brown bears was calculated for all scats during the time period 2015–2018. Per scat the following data were listed: GPS coordinates, start and end date/time at the bear bed, bear ID, bear name, sex (female [F] or male [M]), age, age group (subadult [until 3 years] or adult [from 4 years on]), reproductive class, season of scat deposition, number of years the bear was tracked (the amount [1–4] and consecutive years or not [Cons or NonCons]), scat weight, scat volume, Vf diet items in scat, EDC diet and nutrient composition of the diet (online Dryad repository). The ingredient EDC's were grouped in six primary categories: fruit, insects, vertebrates, natural vegetation (graminoids, equisetopsidae, unidentified vegetation, blueberry bushes, mushrooms and leaves and twigs), anthropogenic vegetation (oats, maize and grain/cereals) and other (miscellaneous [<5 %EDC] and eggs). Reproductive class was divided into the categories adult (4 years and older) lone female, adult male, adult female with yearlings, adult female with cubs of the year, subadult (1–3 years of age) female, and subadult male. Subadult females or males still accompanying their mother were excluded from the dataset. Seasonal division was based on annual pivot points that influence bear diets according to Stenset et al. (2016): spring ranges from den exit to moose calving season (ultimately 20 May) (Swenson et al., 1997), summer ranges from 21st of May until the first berry ripening (31st of July), fall starts on the 1st of August and lasts until late October (Friebe et al., 2001).

### 2.6 Statistics

All dietary ingredient proportions (EDC%) and nutrient concentrations (CP, EE, NfE, TDF, Ash), on DM basis and ME basis, as well as the ratios CP:(EE + NfE) and (CP + NfE):EE on DM and ME basis, were analyzed using linear mixed models (LMM) for the effects and all possible two‐way interactions of the factors year, season and reproductive class using RStudio (R4_0_2), specifying bear id as a random effect to account for non‐independence of scats produced by the same individual. Variable selection was performed using the R ‘GLMERSelect’ function of the R package ‘StatisticalModels’ (https://rdrr.io/github/timnewbold/StatisticalModels/) which performs backward stepwise selection of fixed effects to reach a minimal adequate model containing only variables and interactions statistically significant at the α ≤ 0.05 level. Model residuals were normally distributed, as evidenced by Shapiro–Wilk W values ≥0.95, allowing a Gaussian error distribution. Homogeneity of variance was checked by plotting standardized residuals against fitted values. For post‐hoc identification of significant differences between factors in the linear mixed effects models, we used ‘lsmeans’ from R package ‘emmeans’ (Lenth, 2021), resulting in Tukey‐adjusted *p*‐values. The season spring was omitted from the analysis because it was neither represented in every year nor were sufficient reproductive classes represented in spring.

## 3 RESULTS

A total of 886 scats (325 in 2015, 163 in 2016, 157 in 2017 and 241 in 2018) from 55 bears (36 in 2015, 23 in 2016, 24 in 2017 and 33 in 2018) were analyzed for ingredient and nutrient profiles and ratios (online Dryad repository; Appendix S1: Tables S1 and S2, Figures S1–S3).

### 3.1 Ingredient profiles

In fall diets, the amount of fruit consumed (70.4 ± 37.2% EDC) was always higher than in summer diets (d.f. = 832, *t*‐ratio = 16.72, *p* < .0001), although summer diets also contained a considerable percentage of fruits (22.3 ± 33.3% EDC), except for the year 2015 when fruit contribution was lower than in other study years (6.8 ± 21.0% EDC; vs. 2016: d.f. = 834, *t*‐ratio = −7.805, *p* < .0001, vs. 2017: d.f. = 767, *t*‐ratio = −4.178, *p* = .0002, vs. 2018: d.f. = 731, t‐ratio = −5.663, *p* < .0001). Adult males had a lower fruit content in their diets than subadult females (27.5 ± 37.1 vs. 47.1 ± 44.0% EDC; d.f. = 63.8, *t*‐ratio = −3.533, *p* = .0096). The insect share in the bear diets was highest in summer (30.1% ± 34.7% EDC) compared to fall (5.6 ± 14.8% EDC; d.f. = 835, *t*‐ratio = −9.382, *p* < .0001), with the largest summer–fall difference in the year 2015 (delta 38.2% EDC; d.f. = 841, *t*‐ratio = −12.152, *p* < .0001) and no significant difference in 2017 (delta 9.3% EDC; d.f. = 822, *t*‐ratio = −1.707, *p* = .088). Overall, adult males had the lowest share of insects in their diet (13.6 ± 26.7% EDC) and subadult males the highest share (34.2 ± 38.2% EDC; d.f. = 69.5, *t*‐ratio = −3.468, *p* = .0112). Vertebrates were always higher in the summer diet (21.0 ± 34.6% EDC) compared to the fall diet (3.5 ± 13.3% EDC; d.f. = 851, *t*‐ratio = −8.829, *p* < .0001). Females with cubs of the year had the lowest share of vertebrates in their diet (8.0 ± 18.8% EDC) compared to females with yearlings (23.0 ± 33.6% EDC, d.f. = 835.8, *t*‐ratio = −3.18, *p* = .0193) and adult lone females (15.7 ± 31.7% EDC, d.f. = 421.4, *t*‐ratio = −3.26, *p* = .0153). Natural vegetation in the fall diet was low and constant over the years (6.0 ± 18.4% EDC) compared to higher levels in summer (21.5 ± 30.8% EDC, d.f. = 827, *t*‐ratio = −7.372, *p* < .0001), except for the year 2016 where fall and summer both showed low levels (6.2 ± 14.7 and 7.5 ± 15.7% EDC respectively, d.f. = 819, *t*‐ratio = −0.33, *p* = .74). Anthropogenic vegetation consumption (oats, maize, and grain/cereals) was higher in fall diets (14.2 ± 31.8% EDC) compared to summer diets (4.5 ± 18.6% EDC; d.f. = 843, *t*‐ratio = 4.70, *p* < .0001) and was very high in the fall diet of adult males (43.5 ± 44.8% EDC) compared to all other classes ranging from 3.6% to 14.2% EDC; vs. adult lone female (d.f. = 93.2, *t*‐ratio = 5.094, *p* < .0001), versus adult female with yearlings (d.f. = 261.3, *t*‐ratio = 3.846, *p* = .0021), versus adult female with cubs of the year (d.f. = 132.5, *t*‐ratio = 4.854, *p* < .0001), versus subadult female (d.f. = 111.2, *t*‐ratio = 6.205, *p* < .0001) and versus subadult male (d.f. = 139.6, *t*‐ratio = 4.542, *p* = .0002).

### 3.2 Nutrient profiles

**FIGURE 1:**
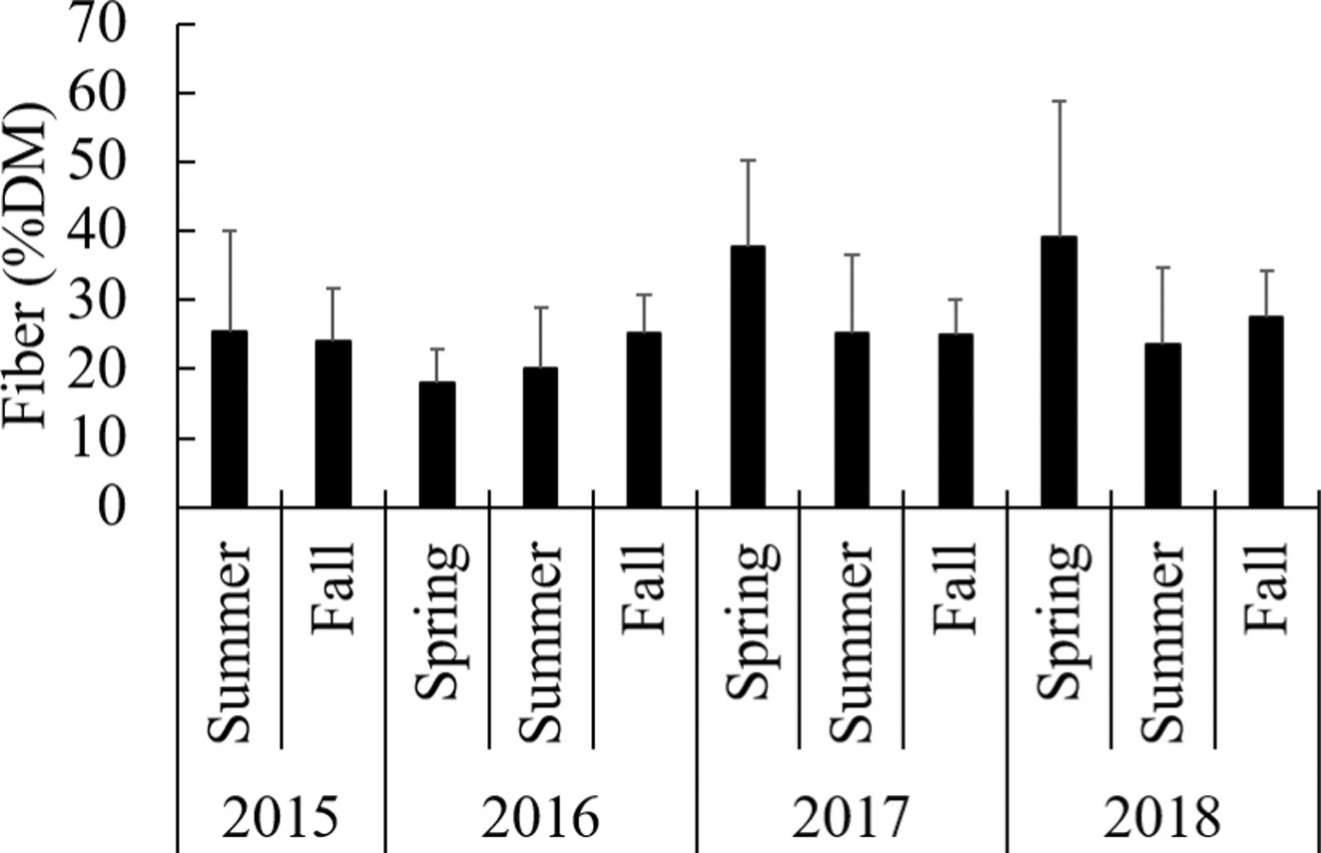
Average fiber concentration (%DM) in the diet of brown bears per year and season. DM, dry matter. The spring season was not included in statistical analyses and statistical output refers only to the comparison of the seasons summer and fall.

The protein content of the ingested diet in summer was almost three times higher (38.0 ± 21.8% DM) compared to the fall diet (12.2 ± 11.8% DM, d.f. = 831, *t*‐ratio = −16.916, *p* < .0001), with the largest summer–fall difference in the year 2015 (delta 34.7%, d.f. = 846, *t*‐ratio = −17.012, *p* < .0001). The dietary fat content was always higher in summer (11.1 ± 6.0% DM) compared to fall (5.3 ± 3.8% DM, d.f. = 833, *t*‐ratio = −13.43, *p* < .0001) with a slight decrease in the difference between summer and fall from 2015 to 2017 (delta 7.6 to delta 3.3%), after which this difference increased again in 2018 (delta 4.7%, all *p* < .0012). The concentration of carbohydrates (NfE fraction) in the summer (27.4 ± 19.8% DM) only reached about half of the carbohydrate concentration of the fall diet (55.3 ± 14.5% DM, d.f. = 838, *t*‐ratio = 18.879, *p* < .0001), and in the year 2015 summer only had one third (19.6 ± 15.3% DM) of the fall carbohydrate concentrations (58.6 ± 15.1% DM, d.f. = 850, *t*‐ratio = 19.767, *p* < .0001). Fiber concentrations were only slightly higher in fall (25.5 ± 6.9% DM) compared to summer (24.0 ± 12.4% DM, d.f. = 830, *t*‐ratio = 2.224, *p* = .0264), with no difference at all in the years 2015 and 2017 (2015: d.f. = 845, *t*‐ratio = −1.295, *p* = .196; 2017: d.f. = 820, *t*‐ratio = −0.085, *p* = .932) (Figure 1). The ash concentration in the summer diet was always higher (5.4 ± 2.8% DM) than in the fall diet (2.8 ± 2.1% DM, d.f. = 832, *t*‐ratio = −12.709, *p* < .0001) with the biggest fall–summer difference in 2015 (delta 4.0%, d.f. = 848, *t*‐ratio = −14.51, *p* < .0001). No significant effects of reproductive class were observed on any of the nutrient concentrations on DM basis. Protein, fat and carbohydrate concentrations on ME basis showed similar annual and seasonal patterns as on DM basis.

### 3.3 Nutrient ratios

**FIGURE 2:**
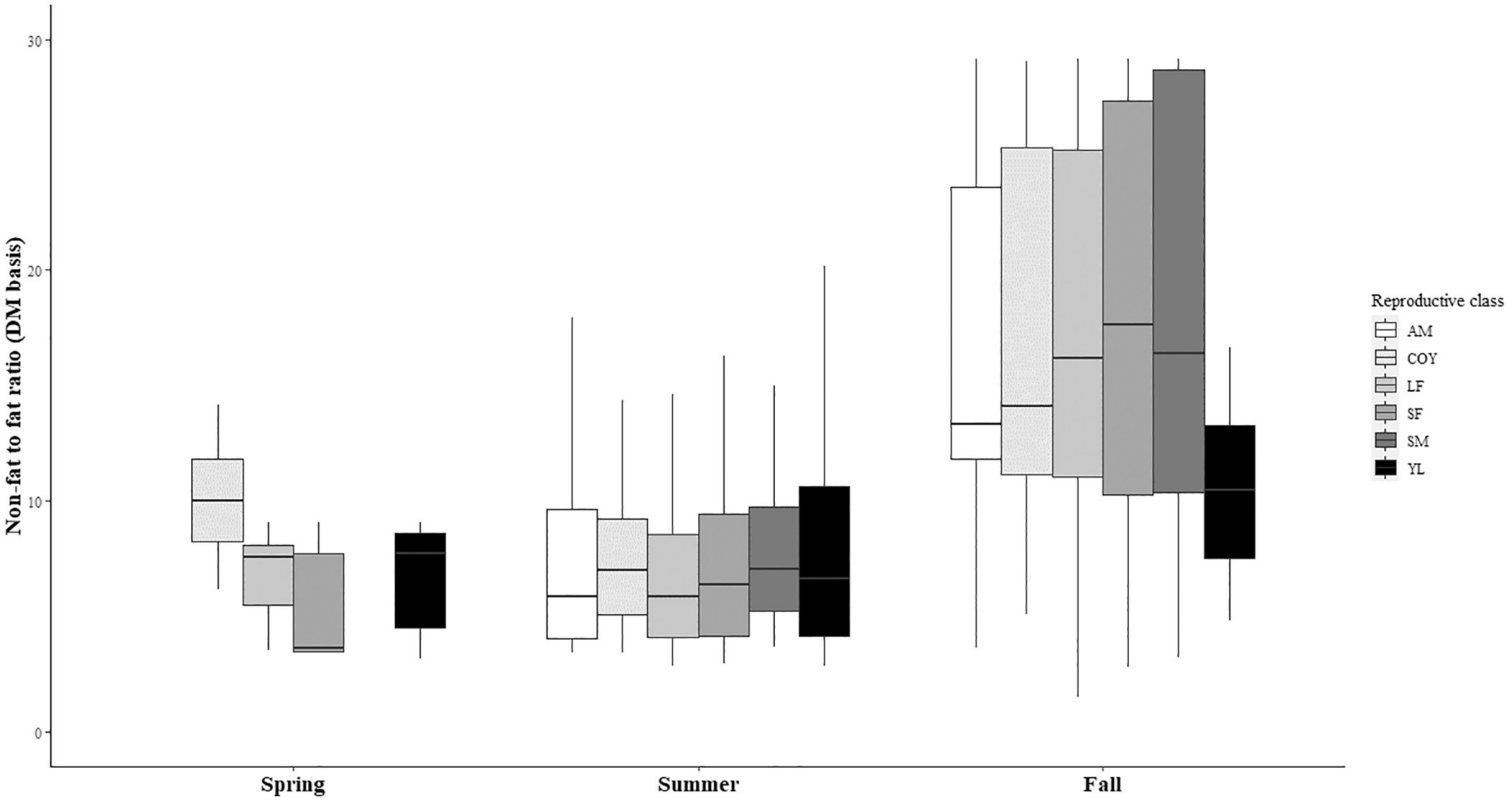
Non‐fat to fat ratio (DM basis) of bear diets per year, season and reproductive class. AM, adult males; COY, females with cubs of the year; DM, dry matter; LF, adult lone females; SF, subadult females; SM, subadult males; YL, females with yearlings. The spring season was not included in statistical analyses and statistical output refers only to the comparison of the seasons summer and fall.

**FIGURE 3:**
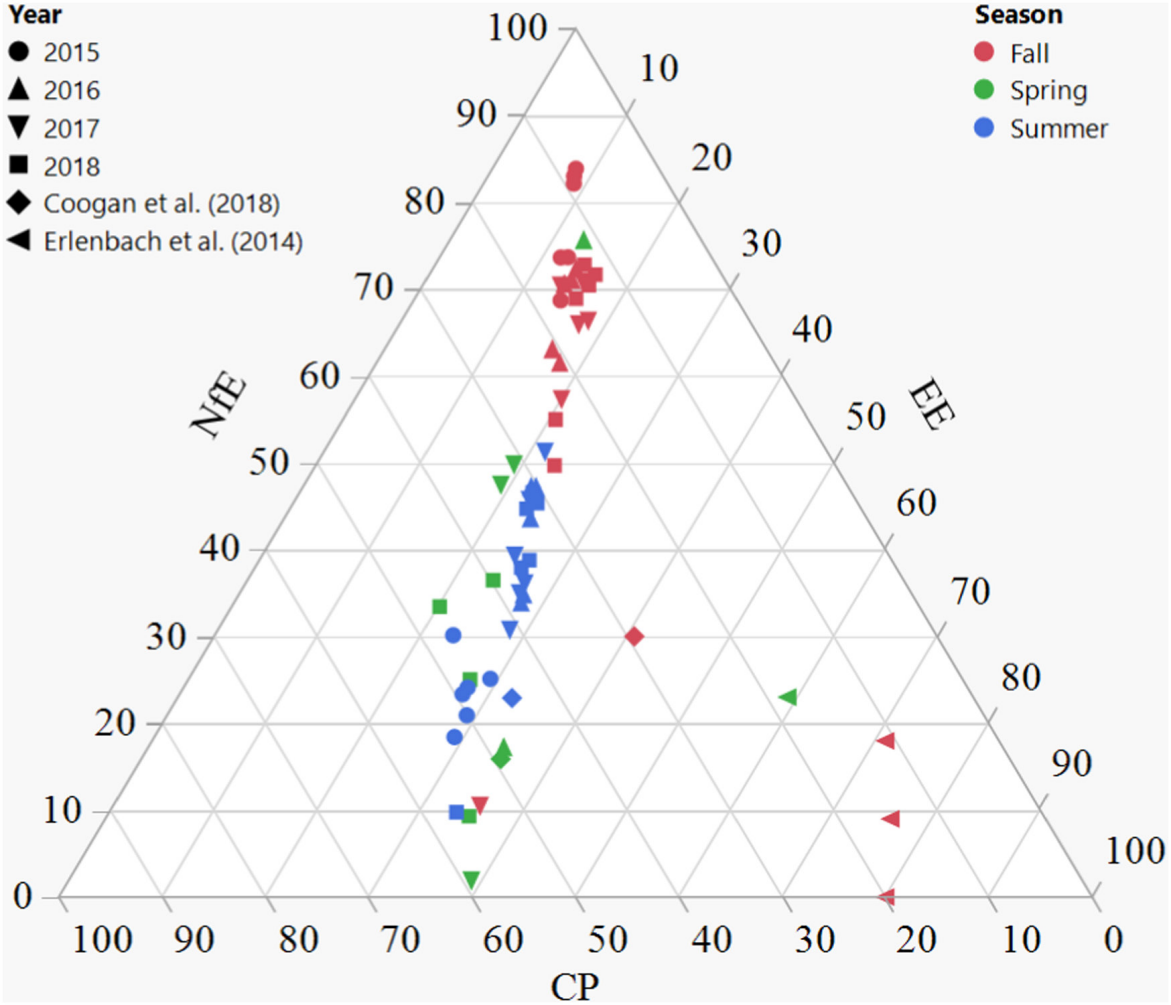
Ternary plot of average dietary CP, EE and NfE concentrations (ME basis) of brown bear diets per year, season and reproductive class. CP, crude protein; EE, ether extract or crude fat; ME, metabolizable energy; NfE, nitrogen‐free extract or digestible carbohydrates. Data from Coogan et al. (2018) are based on bears relying on natural diets. The spring season was not included in statistical analyses and statistical output refers only to the comparison of the seasons summer and fall.

**FIGURE 4:**
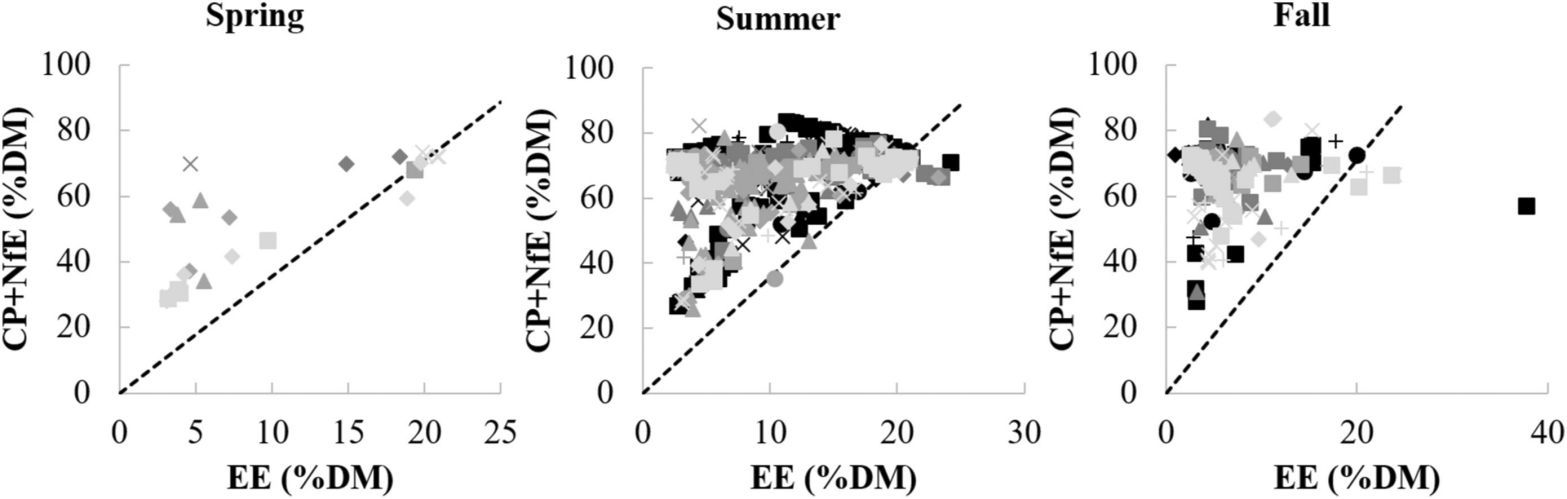
Relationship between EE (%DM) and (CP + NfE) (%DM) of bear diets per year, season and reproductive class. ●, adult males; ■, adult lone females; ▲, females with cubs of the year; ♦, females with yearlings; +, subadult males; ×, subadult females; CP, crude protein; DM, dry matter; EE, ether extract or crude fat; NfE, nitrogen‐free extract or digestible carbohydrates. Black to light gray represent years 2015 (black) to 2018 (lightest gray). The dashed line represents the theoretical minimum of the ratio (CP + NfE):EE to ensure efficient citric acid cycle functioning (3.55) (Appendix S2). The spring season was not included in statistical analyses and statistical output refers only to the comparison of the seasons summer and fall.

The ratio CP:(EE + NfE) on DM basis was low in fall for all years (0.27 ± 0.40) compared to summer, when this ratio was higher (1.3 ± 1.0, d.f. = 835, *t*‐ratio = −16.000, *p* < .0001) with the biggest difference between seasons in the year 2015 (delta 1.5, d.f. = 849, *t*‐ratio = −16.453, *p* < .0001). Similarly, on a ME basis, the CP:(EE + NfE) in fall generally corresponded to the ratio determined by Erlenbach et al. (2014) of 0.2 (0.21 ± 0.24), but was significantly higher in summer (0.80 ± 0.47) in every year (d.f. = 835, *t*‐ratio = −18.840, *p* < .0001), with the biggest difference between seasons again in the year 2015 (delta 0.84, d.f. = 849, *t*‐ratio = −20.106, *p* < .0001, Figure 3). The ratio (CP + NfE):EE on a DM basis was lowest in summer (8.1 ± 5.6) compared to fall (16.9 ± 8.0) with the largest difference between both seasons in the year 2015 (delta 14.4, d.f. = 840, *t*‐ratio = 17.802, *p* < .0001, Figure 2). Reproductive class affected this ratio only in the fall season, with subadult females having a significantly higher ratio (18.3 ± 8.3) compared to females with yearlings (11.8 ± 6.4, d.f. = 625, *t*‐ratio = 3.218, *p* = .0170) and adult males (16.4 ± 7.3, d.f. = 287, *t*‐ratio = −3.938, *p* = .0014). On a ME basis, the (CP + NfE):EE ratio was higher in fall (7.5 ± 3.6) compared to summer (3.6 ± 2.5, d.f. = 839, *t*‐ratio = 14.792, *p* < .0001), with the biggest difference between fall and summer in the year 2015 (delta 6.4, d.f. = 850, *t*‐ratio = 20.144, *p* < .0001). The (CP + NfE):EE ratio only sporadically fell beneath the theoretical 3.55 limit on DM or 1.58 on ME basis (Figure 4; Appendix S2).

## 4 DISCUSSION

This study confirms that Swedish brown bears have a vertebrate‐ and ant‐dominated diet in spring and early summer and a berry‐dominated diet in fall (Stenset et al., 2016; Swenson et al., 1999). Accordingly, diets were protein‐rich in summer and carbohydrate‐rich in fall. Ingredient profiles differed between reproductive classes. However, no difference was found in nutrient profiles, which suggests that bears in different reproductive classes ingest similar nutrient profiles with different ingredients. Swedish brown bears ingest the low protein to non‐protein ratio (0.2) in fall as reported by Robbins et al. (2007); Robbins, Tollefson, et al. (2022); Robbins, Christian, et al. (2022) and Erlenbach et al. (2014) for optimal fat accumulation before hibernation. However, we did not find this optimal ratio during other seasons. We introduced the nutrient ratio non‐fat to fat as metabolically relevant for optimal nutrient intake and its minimal threshold value that would safeguard efficient maintenance metabolism (see below; Appendix S2). Bears managed to ingest nutrients in proportions that never fell beneath the minimal non‐fat to fat threshold. Whenever non‐fat to fat ratios fall beneath the minimal threshold value, this indicates fat accretion and not instantaneous energy use in fall but might point to inefficient metabolism in spring or summer. Fiber made up a constant share of nutrient profiles of bears, raising the question whether this stems from collateral ingestion or rather active selection to safeguard optimal gut health.

### 4.1 Temporal and social variation in ingredients and nutrients

The diet of brown bears in southcentral Sweden can generally be described as protein‐ (and fat‐) dominated in summer and carbohydrate‐dominated in fall. The latter can be considered a logical consequence of the seasonal ingredient intake: a strong presence of vertebrates (predominantly moose) and ants in summer with a gradual increasing share of berries (predominantly bilberry) toward fall (this study, Stenset et al., 2016; Swenson et al., 1999) (see also online Dryad repository). Similarly, in their review of the nutrient composition of brown bear diets worldwide, Coogan et al. (2018) reported an average brown bear diet that was high in protein (and fat) and low in carbohydrate in spring/summer and vice versa in fall, which was attributed to the generally higher degree of carnivory in spring/summer and the growth of fruit in fall (see also Coogan et al., 2014). Numerically, the dominant ingredients in spring were vertebrates (moose) and natural vegetation (mainly graminoids), which was reflected in high dietary protein concentrations in spring. Fruits made up a significant share of the spring diet, which greatly increased the carbohydrate concentration, which can occur when bears consume berries still on bushes from the previous fall (Stenset et al., 2016). Ingredient and nutrient profiles showed only mild, but statistically significant, inter‐annual variation. The most pronounced interannual differences were very low fruit levels (mostly bilberry, Appendix S1) and a very high insect share (mostly the genus Formica, Appendix S1) in the summer diet of 2015 compared to all other years. Consumption of ants has been linked to the absence or low availability of nutritious fruits in the same period (Noyce et al., 1997; Swenson et al., 1999) and ant availability is considered stable between years compared to berry availability, which makes it a steadier food source (Stenset et al., 2016). On a nutrient level, the fruit‐insect switch was visible in the summer of 2015, leading to low carbohydrate and high protein concentrations. Overall, the seasonal and annual variation of ingredients and nutrients found in this study, as those described previously by Stenset et al. (2016) and Coogan et al. (2018) respectively, seem a logical consequence of the seasonal and interannual resource variability (e.g. berry production, vegetation growth, moose calving).

Besides anthropogenic effects, despotism and sexual selected infanticide have been described as important drivers for habitat choice and food resource quality in our study population (Elfström, Zedrosser, Støen, & Swenson, 2014; Steyaert, Reusch, et al., 2013). We found that the diet of females with cubs of the year is somewhat divergent from other demographic groups in terms of ingredients, which may add to the evidence of sexual selected infanticide affecting the diet of the latter (Steyaert, Reusch, et al., 2013). Vertebrates were least present in the diet of females with cubs of the year compared to adult lone females and females with yearlings. Similarly, Elfström, Zedrosser, Jerina, et al. (2014) found that females with cubs of the year exploited slaughter remains less often than other reproductive groups in the Swedish brown bear population. Ben‐David et al. (2004) reported that female brown bears with cubs of the year consumed less salmon as a result of infanticide risk by dominant males in Alaska, USA. Apart from this difference in ingredient intake between females with cubs of the year and other reproductive classes, we found that adult males had lower fruit and insect shares in their diet compared to subadult females and males. However, adult males had higher anthropogenic resources (mainly oats) in the fall diet compared to all other reproductive classes. The heavy reliance of adult males on oats might indicate that adult males substitute berries with oats as carbohydrate source. One would expect anthropogenic food resources to be more present in the diet of subadult bears instead of adult males: subadult bears are often described as more ‘naïve’, avoiding intraspecific predation by adult males, and therefore occurring more frequently near human settlements (Elfström, Davey, Zedrosser, et al., 2014; Elfström, Zedrosser, Jerina, et al., 2014; Elfström, Zedrosser, Støen, & Swenson, 2014). However, in this study area, oats are also used to bait bears for hunting, and bait sites are often sequestered by adult males (Zedrosser et al., 2013). Conclusions for the diet of adult males however, have to be interpreted carefully due the low sample size (6 bears and 41 scats in fall spread over 4 years).

In terms of nutrients, we found no effect of bear reproductive classes on nutrient composition of the ingested diet (DM and ME basis), nor a class x season interaction, which indicates that bear groups obtain similar nutrient intake profiles by means of different ingredients, and thus regardless of foraging strategy. The latter strengthens more recent evidence in ecology in which animals can obtain a similar fitness with differing foraging strategies, for example in Lesser black‐backed gulls (*Larus fuscus*), where the alternative use of marine versus terrestrial foraging habitats incurred similar energetic costs to parents (Sotillo et al., 2019). Additionally, conspecifics inhabiting different geo‐climatic regions can forage for different ingredients and still maintain a similar nutrient intake (*Gorilla beringei* [Rothman et al., 2007]; *Martes martes* [Gazzola & Balestrieri, 2020; Remonti et al., 2016]; *Martes foina* [Gazzola & Balestrieri, 2020]). However, this means we cannot confirm the finding that a ‘diet quality’ discrepancy exists between reproductive classes. One important note on this matter involves how to define ‘diet quality’ for the brown bear. Previous studies defined low diet quality as low fecal protein and high fecal lignified fiber (acid detergent lignin, ADL) (Steyaert, Reusch, et al., 2013). Assuming that protein‐rich prey always equals a high quality diet for an omnivorous carnivore might be too reductive and linked to the observation that bears possess a typical carnivore‐like gut and digestion process (Clauss et al., 2010; Stevens & Hume, 1995). This assumption may be correct for the summer diet (this study, Coogan et al., 2018), but not necessarily for the fall, when low protein concentrations in the diet guarantee optimal metabolism (Erlenbach et al., 2014; Robbins et al., 2007). In addition, previous research often relies on fecal ‘nutrients’ (e.g. Elfström, Zedrosser, Jerina, et al., 2014; Steyaert et al., 2012; Steyaert, Kindberg, et al., 2013) or chemical elements (N and C; Sergiel et al., 2020), where again high protein concentrations (and low lignified fiber) are suggestive for high diet quality. Although this has been proven useful to assess diet quality of herbivore species, the use in carnivores remains questionable (Gálvez Cerón et al., 2015). Fecal nutrients would represent remnants of undigested material or fiber fermentation (undigestible and unfermented protein or carbohydrates), microbial mass and endogenous losses (McDonald et al., 2011), but do not accurately inform us on what the actual nutrient composition of the ingested diet was in carnivores. We would rather recommend calculating nutrient composition (via e.g. EDC calculation [Hewitt & Robbins, 1996]) such as done by Coogan et al. (2018) and Gazzola and Balestrieri (2020), and more so, nutrient ratios as diet quality indicator given their direct link with metabolism.

### 4.2 Nutrient ratios and their metabolic significance

#### 4.2.1 Protein ratio

Erlenbach et al. (2014) reported the optimum ratio of 0.2 protein to non‐protein (on ME basis) from which bears have the most efficient (fat) mass gain (Robbins et al., 2007), ideally in fall, before hibernation. In their experiments with bears in captivity, Erlenbach et al. (2014) even offered a spring diet consisting of salmon, apples and beef fat ad libitum, which led to a selection of ca. 0.2 protein to non‐protein in that season. Additionally, it was suggested that this low protein intake was important to prevent diseases associated with high protein intake (e.g. kidney disease) (Robbins, Christian, et al., 2022; Robbins, Tollefson, et al., 2022; Rode et al., 2021). We found a ratio of ~0.2 in fall with the non‐protein fraction almost entirely consisting of carbohydrates and not fat (70.7 ± 18.3% ME NfE vs. 14.5 ± 7.6% ME EE) (Figure 3) due to the heavy berry reliance in that period (Appendix S1: Table S1, Figure S1), or for adult males a significant share of anthropogenic vegetation (mainly oats, Appendix S1: Table S1, Figure S1) contributing to carbohydrate intake. In southcentral Sweden, there is simply no abundance of ‘fatty resources’ in fall: there are no salmon streams where bears can feed on, as certain North American brown bear populations do in fall (Ben‐David et al., 2004; Fortin et al., 2007; Gende et al., 2001), nor does this area provide fat‐rich hard mast resources, as is the case for some populations in southern Europe (Paralikidis et al., 2010). Compared to the diet choice experiment of Erlenbach et al. (2014), we do not find the 0.2 ratio (ME) numerically in spring (0.9 ± 0.4) nor in summer (0.8 ± 0.5) (Figure 3). In addition, the average nutrient profiles from bears worldwide (Coogan et al., 2018) showed a protein to non‐protein ratio in fall for bears relying on natural resources that is higher than the 0.2 value (Figure 3) as well as on anthropogenic resources, with less variation in the latter. These data are based on geometric means of several brown bear populations worldwide, which would imply that the low protein optimum is dependent on resource availability and not necessarily present in every specific bear population. Similar to our results, Coogan et al. (2018) did not find this optimum in spring or summer for free‐ranging bears (Figure 3). Hence, the question poses itself whether we can perceive this minimal protein optimum as an optimum that bears strive toward throughout the year, or whether it is rather a fall optimum for efficient fat storage (fat storage is indeed most efficient from dietary fat, followed by carbohydrates and protein, respectively [McDonald et al., 2011]), that in its turn depends on population conditions. The reports on natural spring and summer diets, by Coogan et al. (2018) and our own study, as well as the need for high protein diets for lactating bears (López‐Alfaro et al., 2013) also draws into question whether seasonal protein levels higher than 20% actually may be a health hazard (kidney, liver and cardiovascular disease) for bears (Robbins, Christian, et al., 2022; Robbins, Tollefson, et al., 2022; Rode et al., 2021).

Other omnivorous carnivores such as pine martens (*Martes martes*) and stone martens (*Martes foina*) have been observed to forage for an overall high annual protein ratio (ca. 0.79–1.1 [ME basis; Gazzola & Balestrieri, 2020; Remonti et al., 2016]), although seasonality tends to shift this ratio in pine martens: winter and spring diets (high in protein, animal based diet items) typically have a higher ratio compared to summer and fall diets (high in carbohydrates, fruit consumption) (Remonti et al., 2016). As suggested by Remonti et al. (2016), the benefits of using seasonally abundant resources appear species‐ and condition‐specific. The berry‐ and carbohydrate‐rich diet of Swedish brown bears in fall (low protein ratio) appears beneficial for hibernation because of the efficient fat mass gain that has been linked to it (Erlenbach et al., 2014); however, such diets might impact other species differently: coyotes (*Canis latrans*) seem to experience negative effects of summer diets rich in fruits on their survival and fecundity (Tremblay et al., 1998). Protein ratios (ME basis) in the diet of free‐ranging strict carnivores are generally higher than the fall optimum described for brown bears (0.2), but closer to the protein ratio observed in our bears in spring and summer (0.8–0.9): 1.2 for wolves (*Canis lupus*) (Bosch et al., 2015) and 1.1 for feral cats (*Felis silvestris catus*) (Plantinga et al., 2011). Self‐selection studies with domesticated strict carnivores reported a protein ratio of 0.43 for dogs (*Canis familiaris*) (Hewson‐Hughes et al., 2013), 1.1 for cats (*Felis catus*) (Hewson‐Hughes et al., 2011) and 0.54 for mink (*Neovison vison*; with constant carbohydrate 15% ME) (Mayntz et al., 2009). Several strict carnivores are physiologically constrained to use only high protein resources because of the inability to downregulate amino acid catabolic enzymes (e.g. domestic cat; Morris, 2002). Low protein levels in the diet of mink (as low as 15% ME, hence a protein ratio of 0.18) have negative effects on the liver (Damgaard et al., 1998). Other species such as the wolf (Kreeger, 2003) and polar bear (*Ursus maritimus*) (Derocher et al., 1990) have a protein sparing capacity and can cope with periods of low prey availability or famine.

The effects of the dietary protein ratio on hibernation and subsequent reproduction in other hibernating species have been studied to some extent. A study with European hamsters (*Cricetus cricetus*) under human care showed that high protein diets (CP 23, EE 13, NfE 63% ME) led to higher reproductive success in hamsters post‐hibernation compared to high fat diets (CP 15, EE 24, NfE 62%ME), although when we calculate the protein ratio, it is rather similar to the low protein ratio observed in fall for brown bears in both cases (0.30 and 0.17) (Weitten et al., 2018). In European edible dormice (*Glis glis*), the diet in the pre‐hibernating phase mainly consists of fatty seeds (Fietz et al., 2005) and in the primate *Cheirogaleus medius* of carbohydrate‐rich fruits (Fietz & Ganzhorn, 2012), both indicating low dietary protein ratios.

#### 4.2.2 Non‐fat to fat ratio

We evaluated the nutrient ratio (CP + NfE):EE, that is non‐fat to fat ratio, which we suggest as a tool to evaluate whether bears feed for efficient maintenance energy metabolism. The process of combusting nutrients for energy is subject to biochemical limitations that may prevent animals from going for the ‘highest energy resource’ possible. This consideration is based on the function of the citric acid cycle. The citric acid cycle is the final common step in aerobic oxidation of the three nutrients (fat, protein and carbohydrates) or ‘fuels’ that leads to ‘free’ available energy (Mayes & Bender, 2003). In this process, beta‐oxidation of lipogenic energy sources always requires a glucogenic energy source (carbohydrates or protein) for combustion (Akram, 2014; Kohlmeier, 2015). Fatty acids from lipid sources enter the citric acid cycle as acetyl coenzyme A (acetyl CoA). To form citric acid, acetyl CoA needs to react with oxaloacetate, which can only be generated from glucose (carbohydrates), amino acids (protein) or – in the case of animals with fermentative digestion – propionate (Bergman, 1990; McDonald et al., 2011). The inability of fatty acids to form oxaloacetate may thus make dietary fat a limiting nutrient for the metabolism of the individual, hence possibly influencing diet selection. For instance, in some rodents and ruminants, high fat availability (either in the diet or from the mobilization of body fat) in parallel to a lack of glucogenic substrate for the citric acid cycle can lead to ketosis or fatty liver syndrome (Herdt, 2000; Kennedy et al., 2007; Schmid et al., 2020). We calculated the theoretical minimum of the ratio (CP + NfE):EE (see detailed information in Appendix S2) which should reach a value of 3.55 (on DM basis) or 1.58 (on ME basis) in order to ensure efficient citric acid cycle functioning.

The non‐fat to fat ratio of the diet of the Swedish bear population almost never fell beneath this threshold (Figure 4). The ratio did show a marked increase between summer (8.1; DM basis) and fall (16.9) with the largest range for the latter (Figure 2). Bears rely heavily on carbohydrate‐rich berries during fall (this study, Stenset et al. [2016]), and generally ingest less protein and fat than in summer, where high protein (but never as high as the carbohydrate concentrations in fall) and fat concentrations are ingested, which matches the nutrient ratio difference between seasons. Using the data from Coogan et al. (2018) for natural diets, this ratio similarly stayed above the minimal threshold of 1.58 (spring: 1.87, summer: 2.08 and fall: 2.34; ME basis). Using the data from choice experiments of Erlenbach et al. (2014), brown bears ingested diets of a lower (CP + NfE):EE ratio (0.5–1.5 [on DM basis]) whenever the items to choose from allowed it, including the mentioned spring experiment. In other words, the evolved nutrient intake in brown bears is not tuned to an optimal instantaneous use of energy, but rather to the accumulation of fat body reserves, and hence may represent an adaptation to hibernation that hardly affects diet choices in other seasons given the ecologically available options. Similarly, a low ratio (<3.55 DM or <1.58 ME) can be expected in brown bear populations where the fall diet is dominated by fatty hard mast (Paralikidis et al., 2010). This does not necessarily mean inefficient metabolism, but rather a sign of fat accretion in the pre‐hibernating phase where dietary fat is directly stored as body fat, bypassing the citric acid cycle (Bhagavan, 2002; McDonald et al., 2011). If, however, low non‐fat to fat ratios beneath the theoretical threshold would occur in spring or summer, where fat accretion is not really a ‘necessary metabolic outcome’, this might lead to inefficient metabolism. It seems that the Swedish bear population ingested diets – whether by active selection or ecological opportunity – that never exerted a lack of glucogenic substrate.

Note that dietary fat concentrations varied in a smaller range under lower limits (min. 2.4 – max. 24.2% DM; min 7.2 – max. 44.7% ME; without outlier value) compared to protein (min. 3.7 – max. 73.0% DM; min 4.6 – max. 63.7% ME) and carbohydrates (min. 1.1 – max. 72.5% DM; min 0.88 – max. 86.2% ME) (Figure 4), which could point to a limitation of fat intake. The latter might just emerge from ecological circumstances, that is, there are no food resources available with higher fat concentrations. Fat content across all diet items ranged from min. 2.5 to max. 24.3% DM (vertebrate prey) and 7.2 to max. 45.1% ME, without taking into account the outlier of bird eggs for which the EE equals 39.9% DM or 61.7% ME. As such, the limit on fat intake reflects the maximal EE values of food resources, hence it seems that there was no active selection for an ‘ideal’ dietary fat content.

### 4.3 Dietary fiber in the brown bear's diet

Nutrient profiles of the Swedish bear population in southcentral Sweden always included considerable and constant amounts of fiber throughout the years and seasons (Figure 1). There was only a slight significant difference between fall (25.5% DM) and summer (24.0% DM). Numerically, the highest fiber intake concentrations were observed in spring (36.5% DM) (Figure 1). These seasonal concentrations seem not surprising, given the fact that typical food items in the diet of brown bears contain considerable amounts of plant as well as animal fiber: berries (23.2%–45.6% DM); insects (2.7%–26.6% DM); vertebrates (6.3%–17.3% DM); natural vegetation (13.3%–59.4% DM); anthropogenic vegetation (11.8%–24.7% DM). For several other omnivorous and herbivorous species, it has become apparent that an adequate dietary fiber profile safeguards intestinal as well as systemic health through modulation of microbial fermentation patterns in the gut (Macfarlane et al., 1992; Macfarlane & Gibson, 1995; Wong et al., 2006). More recently, there is evidence that this similarly holds true for carnivores (Depauw et al., 2012, 2013; Whitehouse‐Tedd et al., 2015).

Given the overall considerable amount of fiber in nutrient profiles of bears (25.0% DM) (Figure 1), the question arises whether or not the relevance of this nutrient has been underestimated so far for bears or for carnivores in general. Can we discuss optimum nutrient profiles only based on digestible protein, digestible carbohydrates, and fat? The rather high and constant fiber concentration could just be the mere result of ‘collateral ingestion’, or the observed intake concentrations might represent a threshold value that bears ingest to ensure optimal gut health and motility without impairing digestible energy consumption (Burrows et al., 1982; Kienzle et al., 2006).

One final note on fiber intake concentrations and the inclusion of animal fiber in the latter is that including animal fiber (i.e. the indigestible proteinaceous fractions in animal matter [prey] such as tendons, bone, hair [Depauw et al., 2013]) will have an effect on protein concentration in nutrient profiles of bears, given its proteinaceous nature that is now erroneously included in the (digestible) protein contents of feeds. The topic of fiber requires further attention in the future and the formulation of minimal fiber requirements might be necessary. We further encourage authors to report dietary fiber concentrations (including animal fiber) in nutrient profiles of omnivorous and strict carnivores and this to allow comparison among species.

### 4.4 Conclusion

Our study demonstrates that free‐ranging bears can obtain similar nutrient intakes regardless of foraging strategy or social dynamics. Nutrient intake seems to be tuned to an optimal preparation for hibernation in fall (low protein ratio for efficient fat mass gain); however, in other seasons, other processes – metabolic or ecologic – might drive nutrient selection. Fiber had a considerable share in the diets of brown bears, hence should be given more attention in the future.

## AUTHOR CONTRIBUTIONS

**Annelies De Cuyper:** Conceptualization (equal); data curation (lead); formal analysis (equal); funding acquisition (equal); investigation (equal); methodology (equal); project administration (lead); resources (equal); validation (equal); visualization (equal); writing – original draft (lead); writing – review and editing (equal). **Diederik Strubbe:** Conceptualization (equal); data curation (equal); formal analysis (lead); investigation (equal); methodology (equal); software (lead); validation (equal); visualization (equal); writing – review and editing (equal). **Marcus Clauss:** Conceptualization (equal); data curation (equal); formal analysis (equal); investigation (equal); methodology (equal); supervision (equal); validation (equal); writing – review and editing (equal). **Luc Lens:** Conceptualization (equal); funding acquisition (equal); investigation (equal); methodology (equal); project administration (equal); resources (equal); supervision (equal); validation (equal); writing – review and editing (equal). **Andreas Zedrosser:** Data curation (equal); investigation (equal); methodology (equal); project administration (equal); resources (equal); supervision (equal); validation (equal); writing – review and editing (equal). **Sam Steyaert:** Data curation (equal); investigation (equal); methodology (equal); supervision (equal); validation (equal); writing – review and editing (equal). **Leen Verbist:** Conceptualization (equal); data curation (equal); methodology (equal); validation (equal); writing – review and editing (equal). **Geert P. J. Janssens:** Conceptualization (equal); formal analysis (equal); funding acquisition (equal); investigation (equal); methodology (equal); project administration (equal); resources (equal); supervision (lead); validation (equal); visualization (equal); writing – review and editing (equal).

## CONFLICT OF INTEREST STATEMENT

The authors declare that they have no conflict of interest.

## Supporting information


Appendix S1.

Appendix S2.

## Data Availability

Data for: Ingredient and nutrient composition of brown bear diets: Dryad doi: 10.5061/dryad.dbrv15f64.
