# Supplementary material for: Nutrient intake and its possible drivers in free‐ranging European brown bears (Ursus arctos arctos)
Source: Ecol Evol. 2023 May 30;13(5):e10156. doi: 10.1002/ece3.10156 (PMC10227639; doi:10.1002/ece3.10156)
Supplement: Supplementary file 1 — Appendix S1. [file ECE3-13-e10156-s001.docx]

**Nutrient intake and its possible drivers in free-ranging European brown bears (*Ursus arctos arctos*)**

Annelies De Cuyper^1^, Diederik Strubbe^2^, Marcus Clauss^3^, Luc Lens^2^, Andreas Zedrosser^4,5^, Sam Steyaert^6^, Leen Verbist^7^, Geert P. J. Janssens^1^

*^1^Department of Veterinary and Biosciences, Faculty of Veterinary Medicine, Ghent University, Merelbeke, Belgium*

*^2^Terrestrial Ecology Unit, Department of Biology, Faculty of Sciences, Ghent University, Ghent, Belgium*

*^3^Clinic for Zoo Animals, Exotic Pets and Wildlife, Vetsuisse Faculty, University of Zurich, Zurich, Switzerland*

*^4^Department of Natural Sciences and Environmental Health, Faculty of Technology, Natural Sciences and Maritime Sciences, University of South-Eastern Norway, Bø, Norway*

*^5^Institute for Wildlife Biology and Game Management, University for Natural Resources and Life Sciences, Vienna, Austria*

*^6^Faculty of Biosciences and Aquaculture, Nord University, Steinkjer, Norway*

*^7^Onderzoekskern Salto, Odisee Hogeschool, Campus Sint-Niklaas, Sint-Niklaas, Belgium*

**Appendix 1**

TABLES

**Table A1 Average percentage of estimated dietary content (EDC) of ingredients per year, season and reproductive class in free-ranging Swedish brown bears.**

|  |  |  |  | % EDC | | | | | |
| --- | --- | --- | --- | --- | --- | --- | --- | --- | --- |
| Year | Season | Class | N (scats) | Fruits | Insects | Vertebrates | Natural vegetation | Anthropogenic  Vegetation | Other |
| 2015 | Fall | Adult males | 27 | 43.7 | 4.3 | 3.5 | 6.9 | 41.6 | 0.0 |
|  |  | Adult lone females | 60 | 74.9 | 6.6 | 0.4 | 6.4 | 10.1 | 1.6 |
|  |  | Females with COY | 12 | 87.8 | 3.1 | 0.0 | 0.8 | 8.2 | 0.0 |
|  |  | Females with YL | 2 | 97.8 | 2.2 | 0.0 | 0.0 | 0.0 | 0.0 |
|  |  | Subadult males | 11 | 76.4 | 11.5 | 0.0 | 5.1 | 6.9 | 0.0 |
|  |  | Subadult females | 13 | 96.0 | 2.9 | 0.0 | 1.0 | 0.0 | 0.0 |
|  | Summer | Adult males | 33 | 3.5 | 24.9 | 32.0 | 22.7 | 17.0 | 0.0 |
|  |  | Adult lone females | 84 | 8.2 | 41.9 | 20.6 | 26.4 | 2.9 | 0.0 |
|  |  | Females with COY | 18 | 6.0 | 52.9 | 12.4 | 28.3 | 0.4 | 0.0 |
|  |  | Females with YL | 11 | 3.7 | 45.8 | 32.9 | 17.6 | 0.0 | 0.0 |
|  |  | Subadult males | 19 | 13.7 | 58.0 | 5.3 | 23.0 | 0.0 | 0.0 |
|  |  | Subadult females | 35 | 4.0 | 54.2 | 16.0 | 25.8 | 0.0 | 0.0 |
| 2016 | Fall | Adult males | 10 | 50.2 | 1.3 | 2.8 | 2.1 | 43.5 | 0.0 |
|  |  | Adult lone females | 30 | 72.8 | 6.5 | 3.9 | 3.2 | 13.6 | 0.0 |
|  |  | Females with COY | 13 | 68.8 | 9.6 | 4.4 | 17.1 | 0.0 | 0.0 |
|  |  | Females with YL | 4 | 74.9 | 4.6 | 16.8 | 3.5 | 0.2 | 0.0 |
|  |  | Subadult females | 8 | 78.0 | 10.9 | 0.9 | 6.2 | 4.0 | 0.0 |
|  | Spring | Females with YL | 2 | 25.8 | 2.5 | 70.5 | 1.2 | 0.0 | 0.0 |
|  |  | Subadult females | 1 | 87.5 | 6.7 | 0.9 | 4.9 | 0.0 | 0.0 |
|  | Summer | Adult males | 8 | 41.7 | 16.2 | 25.7 | 7.7 | 8.6 | 0.0 |
|  |  | Adult lone females | 39 | 36.9 | 28.5 | 27.3 | 4.5 | 2.2 | 0.6 |
|  |  | Females with COY | 24 | 49.4 | 21.3 | 14.0 | 15.0 | 0.2 | 0.0 |
|  |  | Females with YL | 16 | 37.3 | 6.8 | 44.6 | 6.5 | 4.5 | 0.2 |
|  |  | Subadult females | 8 | 45.5 | 26.6 | 13.9 | 1.7 | 12.3 | 0.0 |
| 2017 | Fall | Adult males | 3 | 46.8 | 8.1 | 0.0 | 2.0 | 43.1 | 0.0 |
|  |  | Adult lone females | 10 | 71.7 | 21.2 | 3.8 | 3.3 | 0.0 | 0.0 |
|  |  | Females with COY | 10 | 75.1 | 7.8 | 1.1 | 2.2 | 13.8 | 0.0 |
|  |  | Females with YL | 4 | 61.8 | 11.6 | 0.0 | 2.0 | 24.3 | 0.3 |
|  |  | Subadult males | 1 | 5.5 | 34.3 | 47.1 | 13.1 | 0.0 | 0.0 |
|  |  | Subadult females | 4 | 50.6 | 6.6 | 0.8 | 2.9 | 39.2 | 0.0 |
|  | Spring | Adult lone females | 1 | 0.0 | 0.0 | 90.2 | 9.8 | 0.0 | 0.0 |
|  |  | Females with COY | 3 | 36.3 | 6.3 | 4.5 | 53.0 | 0.0 | 0.0 |
|  |  | Females with YL | 3 | 31.1 | 6.1 | 7.8 | 55.0 | 0.0 | 0.0 |
|  | Summer | Adult males | 10 | 32.8 | 9.1 | 11.0 | 35.5 | 9.7 | 1.9 |
|  |  | Adult lone females | 19 | 15.8 | 25.4 | 27.5 | 25.5 | 5.6 | 0.2 |
|  |  | Females with COY | 41 | 19.4 | 27.1 | 8.4 | 40.7 | 3.8 | 0.6 |
|  |  | Females with YL | 24 | 39.1 | 18.9 | 14.2 | 27.6 | 0.0 | 0.2 |
|  |  | Subadult males | 10 | 18.7 | 35.3 | 9.0 | 35.2 | 0.6 | 1.1 |
|  |  | Subadult females | 14 | 24.2 | 11.1 | 39.7 | 15.9 | 9.0 | 0.1 |
| 2018 | Fall | Adult males | 1 | 4.6 | 0.0 | 0.0 | 0.0 | 95.4 | 0.0 |
|  |  | Adult lone females | 44 | 69.2 | 0.1 | 6.1 | 1.2 | 23.4 | 0.0 |
|  |  | Females with COY | 15 | 75.5 | 0.0 | 8.7 | 0.1 | 15.7 | 0.0 |
|  |  | Females with YL | 7 | 44.1 | 28.2 | 3.8 | 10.5 | 13.4 | 0.0 |
|  |  | Subadult males | 7 | 54.1 | 2.5 | 17.6 | 25.0 | 0.9 | 0.0 |
|  |  | Subadult females | 43 | 79.6 | 2.3 | 3.3 | 13.4 | 1.3 | 0.0 |
|  | Spring | Adult lone females | 5 | 19.9 | 3.8 | 6.3 | 68.8 | 0.0 | 1.3 |
|  |  | Females with COY | 1 | 0.0 | 0.8 | 0.0 | 98.2 | 0.0 | 0.9 |
|  |  | Females with YL | 7 | 2.3 | 5.9 | 23.0 | 67.9 | 0.0 | 0.9 |
|  |  | Subadult females | 5 | 0.0 | 10.1 | 68.9 | 20.1 | 0.0 | 0.8 |
|  | Summer | Adult males | 3 | 3.6 | 31.1 | 63.1 | 1.5 | 0.3 | 0.4 |
|  |  | Adult lone females | 36 | 35.3 | 14.7 | 34.9 | 10.1 | 3.7 | 1.3 |
|  |  | Females with COY | 10 | 37.9 | 29.3 | 6.2 | 24.3 | 0.6 | 1.8 |
|  |  | Females with YL | 24 | 20.3 | 20.0 | 23.2 | 17.4 | 17.5 | 1.7 |
|  |  | Subadult males | 4 | 43.8 | 35.6 | 5.3 | 13.1 | 0.0 | 2.2 |
|  |  | Subadult females | 29 | 38.0 | 20.1 | 13.0 | 21.5 | 5.6 | 1.8 |
| Statistics | P-values | Year |  | <0.001 | <0.001 | - | 0.0022 | - | - |
|  |  | Season |  | <0.001 | <0.001 | <0.001 | <0.001 | <0.001 | - |
|  |  | Class |  | 0.021 | 0.021 | 0.0025 | - | <0.001 | - |
|  |  | Year*Season |  | <0.001 | <0.001 | - | <0.001 | - | - |
|  |  | Year*Class |  | - | - | - | - | - | - |
|  |  | Season*Class |  | - | - | - | - | <0.001 | - |

EDC = estimated dietary content; COY = cubs of the year; YL = yearlings. The spring season was not included in statistical analyses and statistical output refers only to the comparison of the seasons summer and fall.

**Table A2 Average nutrient composition and ratios (% DM and ME) of brown bear diets per year, season and reproductive class**

|  |  |  |  | % DM* | | | | | | | | | % ME | | | | | |
| --- | --- | --- | --- | --- | --- | --- | --- | --- | --- | --- | --- | --- | --- | --- | --- | --- | --- | --- |
| Year | Season | Class | N (scats) | CP | EE | NfE | TDF | Ash | ME (kJ/100gDM) | **(CP+NfE):EE** | **CP:(NfE+EE)** | CP | | EE | NfE | **(CP+NfE):EE** | **CP:(NfE+EE)** |  |
| 2015 | Fall | Adult males | 27 | 13.8 | 5.2 | 53.6 | 25.2 | 3.0 | 1322.3 | 17.2 | 0.3 | 17.1 | | 14.2 | 68.7 | 7.6 | 0.3 |  |
|  |  | Adult lone females | 60 | 11.2 | 4.7 | 57.8 | 24.4 | 2.5 | 1329.2 | 21.0 | 0.2 | 13.9 | | 12.4 | 73.7 | 9.3 | 0.2 |  |
|  |  | Females with COY | 12 | 7.5 | 3.2 | 65.7 | 22.0 | 1.7 | 1344.1 | 25.3 | 0.1 | 9.1 | | 8.8 | 82.2 | 11.3 | 0.1 |  |
|  |  | Females with YL | 2 | 6.4 | 2.9 | 66.4 | 23.3 | 1.7 | 1324.0 | 25.9 | 0.1 | 8.0 | | 8.1 | 83.9 | 11.5 | 0.1 |  |
|  |  | Subadult males | 11 | 13.0 | 4.6 | 57.6 | 23.1 | 2.4 | 1351.2 | 21.4 | 0.3 | 14.6 | | 11.8 | 73.6 | 9.5 | 0.2 |  |
|  |  | Subadult females | 13 | 6.9 | 2.9 | 65.4 | 23.3 | 1.8 | 1319.5 | 25.5 | 0.1 | 8.0 | | 8.1 | 83.9 | 11.4 | 0.1 |  |
|  | Summer | Adult males | 33 | 43.2 | 12.7 | 20.2 | 25.7 | 6.2 | 1535.5 | 6.3 | 1.6 | 45.7 | | 29.2 | 25.1 | 2.8 | 1.0 |  |
|  |  | Adult lone females | 84 | 44.8 | 11.7 | 19.7 | 25.9 | 6.8 | 1519.8 | 7.1 | 1.7 | 48.4 | | 27.5 | 24.1 | 3.1 | 1.0 |  |
|  |  | Females with COY | 18 | 45.7 | 11.7 | 18.8 | 28.6 | 6.1 | 1517.9 | 6.8 | 1.6 | 49.3 | | 27.4 | 23.3 | 3.0 | 1.0 |  |
|  |  | Females with YL | 11 | 54.3 | 13.5 | 14.9 | 23.4 | 6.1 | 1665.4 | 6.2 | 2.1 | 52.5 | | 29.0 | 18.4 | 2.8 | 1.2 |  |
|  |  | Subadult males | 19 | 43.8 | 9.7 | 25.8 | 25.0 | 6.3 | 1527.9 | 8.3 | 1.5 | 46.8 | | 23.1 | 30.1 | 3.7 | 1.0 |  |
|  |  | Subadult females | 35 | 48.9 | 12.9 | 17.4 | 23.2 | 6.4 | 1596.4 | 6.1 | 1.8 | 50.1 | | 29.0 | 20.9 | 2.7 | 1.1 |  |
| 2016 | Fall | Adult males | 10 | 11.1 | 4.9 | 57.3 | 24.6 | 2.6 | 1329.5 | 14.8 | 0.2 | 13.8 | | 13.9 | 72.3 | 6.6 | 0.2 |  |
|  |  | Adult lone females | 30 | 12.4 | 5.2 | 57.3 | 24.2 | 2.4 | 1358.7 | 15.1 | 0.2 | 14.8 | | 14.1 | 71.0 | 6.7 | 0.2 |  |
|  |  | Females with COY | 13 | 15.3 | 5.5 | 48.8 | 29.1 | 3.4 | 1279.9 | 12.9 | 0.4 | 20.7 | | 16.2 | 63.0 | 5.8 | 0.3 |  |
|  |  | Females with YL | 4 | 18.5 | 7.0 | 51.8 | 22.5 | 3.2 | 1440.21 | 12.20 | 0.3 | 20.8 | | 17.7 | 61.5 | 5.4 | 0.3 |  |
|  |  | Subadult females | 8 | 12.9 | 4.9 | 57.0 | 24.3 | 2.5 | 1353.2 | 14.8 | 0.2 | 15.8 | | 13.6 | 70.6 | 6.6 | 0.2 |  |
|  | Spring | Females with YL | 2 | 52.6 | 16.6 | 18.3 | 15.4 | 5.4 | 1811.4 | 4.3 | 1.6 | 48.3 | | 34.4 | 17.3 | 1.9 | 1.0 |  |
|  |  | Subadult females | 1 | 9.2 | 4.6 | 60.8 | 23.5 | 2.2 | 1343.7 | 15.1 | 0.1 | 11.4 | | 13.0 | 75.6 | 6.7 | 0.1 |  |
|  | Summer | Adult males | 8 | 32.6 | 10.5 | 36.1 | 22.0 | 4.1 | 1545.1 | 10.2 | 1.0 | 32.6 | | 23.8 | 43.6 | 4.5 | 0.6 |  |
|  |  | Adult lone females | 39 | 40.0 | 12.9 | 30.6 | 17.6 | 4.3 | 1664.7 | 7.3 | 1.2 | 38.3 | | 27.8 | 33.9 | 3.3 | 0.7 |  |
|  |  | Females with COY | 24 | 28.1 | 9.0 | 38.1 | 23.8 | 4.1 | 1444.6 | 10.2 | 0.8 | 30.7 | | 22.1 | 47.2 | 4.5 | 0.5 |  |
|  |  | Females with YL | 16 | 38.9 | 12.6 | 29.1 | 20.7 | 5.0 | 1611.8 | 7.9 | 1.3 | 37.6 | | 27.5 | 34.8 | 3.5 | 0.8 |  |
|  |  | Subadult females | 8 | 30.3 | 9.9 | 42.1 | 18.1 | 3.3 | 1583.9 | 9.9 | 0.7 | 30.2 | | 22.5 | 47.2 | 4.4 | 0.5 |  |
| 2017 | Fall | Adult males | 3 | 13.1 | 4.8 | 56.7 | 23.9 | 2.5 | 1348.3 | 15.1 | 0.2 | 16.2 | | 13.3 | 70.4 | 6.7 | 0.2 |  |
|  |  | Adult lone females | 10 | 20.1 | 7.7 | 47.7 | 23.4 | 2.7 | 1425.9 | 9.9 | 0.4 | 22.7 | | 20.0 | 57.3 | 4.4 | 0.3 |  |
|  |  | Females with COY | 10 | 11.2 | 5.4 | 56.1 | 26.2 | 2.2 | 1328.1 | 13.6 | 0.2 | 13.9 | | 15.3 | 70.9 | 6.0 | 0.2 |  |
|  |  | Females with YL | 4 | 13.5 | 6.1 | 52.5 | 26.6 | 2.5 | 1330.8 | 11.2 | 0.2 | 16.7 | | 17.3 | 66.0 | 5.0 | 0.2 |  |
|  |  | Subadult males | 1 | 58.0 | 16.9 | 11.3 | 15.2 | 6.1 | 1796.8 | 4.1 | 2.1 | 54.0 | | 35.5 | 10.5 | 1.8 | 1.2 |  |
|  |  | Subadult females | 4 | 12.4 | 6.3 | 52.0 | 27.4 | 2.6 | 1314.8 | 10.3 | 0.2 | 15.6 | | 18.0 | 66.4 | 4.6 | 0.2 |  |
|  | Spring | Adult lone females | 1 | 66.1 | 19.3 | 2.1 | 16.4 | 6.6 | 1868.1 | 3.5 | 3.1 | 59.1 | | 38.9 | 1.9 | 1.6 | 1.4 |  |
|  |  | Females with COY | 3 | 17.3 | 4.9 | 31.8 | 40.9 | 6.1 | 1005.7 | 10.5 | 0.7 | 31.0 | | 19.1 | 49.8 | 4.6 | 0.5 |  |
|  |  | Females with YL | 3 | 19.5 | 5.1 | 29.4 | 41.8 | 6.5 | 1006.8 | 10.7 | 0.7 | 33.5 | | 19.0 | 47.5 | 4.7 | 0.6 |  |
|  | Summer | Adult males | 10 | 21.6 | 7.6 | 39.5 | 28.6 | 4.5 | 1308.2 | 10.5 | 0.7 | 27.3 | | 21.4 | 51.3 | 4.7 | 0.5 |  |
|  |  | Adult lone females | 19 | 40.2 | 12.3 | 27.0 | 20.8 | 4.9 | 1585.1 | 6.4 | 1.3 | 41.0 | | 28.2 | 30.8 | 2.8 | 0.8 |  |
|  |  | Females with COY | 41 | 29.6 | 9.1 | 30.5 | 28.5 | 5.0 | 1346.2 | 7.9 | 0.9 | 36.2 | | 24.5 | 39.3 | 3.5 | 0.6 |  |
|  |  | Females with YL | 24 | 27.0 | 8.7 | 36.7 | 25.9 | 4.7 | 1393.2 | 9.6 | 0.8 | 31.6 | | 22.7 | 45.7 | 4.3 | 0.6 |  |
|  |  | Subadult males | 10 | 33.7 | 11.0 | 29.7 | 23.7 | 4.7 | 1475.0 | 7.1 | 1.0 | 36.9 | | 27.0 | 36.1 | 3.1 | 0.6 |  |
|  |  | Subadult females | 14 | 39.6 | 12.3 | 29.3 | 19.7 | 4.7 | 1615.9 | 8.0 | 1.5 | 37.9 | | 27.1 | 35.0 | 3.6 | 0.8 |  |
| 2018 | Fall | Adult males | 1 | 12.3 | 5.5 | 54.7 | 24.6 | 2.9 | 1327.0 | 12.2 | 0.2 | 15.5 | | 15.6 | 68.9 | 5.4 | 0.2 |  |
|  |  | Adult lone females | 44 | 10.7 | 5.9 | 54.9 | 26.8 | 2.5 | 1317.1 | 14.9 | 0.2 | 12.3 | | 16.1 | 71.6 | 6.6 | 0.2 |  |
|  |  | Females with COY | 15 | 11.5 | 5.9 | 55.9 | 25.3 | 2.5 | 1348.0 | 14.6 | 0.2 | 13.5 | | 16.1 | 70.4 | 6.5 | 0.2 |  |
|  |  | Females with YL | 7 | 25.3 | 8.4 | 38.0 | 29.3 | 4.3 | 1375.4 | 7.9 | 0.7 | 27.2 | | 23.1 | 49.7 | 3.5 | 0.5 |  |
|  |  | Subadult males | 7 | 19.8 | 7.6 | 41.1 | 28.0 | 5.6 | 1304.3 | 13.7 | 0.6 | 24.5 | | 20.6 | 55.0 | 6.1 | 0.4 |  |
|  |  | Subadult females | 43 | 9.8 | 4.9 | 54.0 | 28.5 | 3.5 | 1251.2 | 17.5 | 0.2 | 12.8 | | 14.5 | 72.8 | 7.8 | 0.2 |  |
|  | Spring | Adult lone females | 5 | 19.0 | 5.6 | 19.2 | 48.6 | 7.5 | 846.9 | 7.5 | 1.0 | 39.7 | | 23.8 | 36.5 | 3.3 | 0.8 |  |
|  |  | Females with COY | 1 | 16.7 | 3.2 | 12.0 | 57.8 | 9.4 | 601.6 | 8.9 | 1.1 | 46.5 | | 20.1 | 33.4 | 4.0 | 0.9 |  |
|  |  | Females with YL | 7 | 30.8 | 8.6 | 11.5 | 42.5 | 8.8 | 1031.2 | 6.8 | 1.5 | 47.7 | | 27.3 | 25.0 | 3.0 | 0.9 |  |
|  |  | Subadult females | 5 | 58.7 | 17.0 | 5.2 | 21.3 | 6.8 | 1708.1 | 4.7 | 2.6 | 55.7 | | 35.0 | 9.3 | 2.1 | 1.3 |  |
|  | Summer | Adult males | 3 | 64.5 | 17.2 | 10.4 | 14.4 | 5.8 | 1899.0 | 4.9 | 2.5 | 56.6 | | 33.6 | 9.8 | 2.2 | 1.3 |  |
|  |  | Adult lone females | 36 | 37.1 | 11.6 | 29.7 | 21.4 | 4.6 | 1553.2 | 10.7 | 1.4 | 36.3 | | 25.8 | 37.9 | 4.8 | 0.8 |  |
|  |  | Females with COY | 10 | 27.0 | 8.5 | 34.5 | 27.0 | 4.9 | 1349.6 | 11.9 | 0.9 | 32.4 | | 22.9 | 44.7 | 5.3 | 0.6 |  |
|  |  | Females with YL | 24 | 33.8 | 11.0 | 30.2 | 23.1 | 5.5 | 1485.2 | 8.1 | 1.1 | 35.1 | | 26.1 | 38.8 | 3.6 | 0.7 |  |
|  |  | Subadult males | 4 | 28.0 | 9.2 | 37.2 | 23.9 | 3.9 | 1437.3 | 8.4 | 0.8 | 31.0 | | 23.6 | 45.4 | 3.7 | 0.5 |  |
|  |  | Subadult females | 29 | 26.3 | 8.8 | 35.4 | 26.3 | 4.9 | 1363.7 | 11.3 | 0.9 | 30.9 | | 22.6 | 46.6 | 5.0 | 0.6 |  |
| Statistics | P-values | Year |  | 0.0027 | - | 0.015 | 0.056 | <0.001 | - | <0.001 | <0.001 | <0.001 | | - | 0.017 | <0.001 | <0.001 |  |
|  |  | Season |  | <0.001 | <0.001 | <0.001 | 0.027 | <0.001 | - | <0.001 | <0.001 | <0.001 | | <0.001 | <0.001 | <0.001 | <0.001 |  |
|  |  | Class |  | - | - | - | - | - | - | 0.0049 | - | - | | - | - | - | - |  |
|  |  | Year*Season |  | <0.001 | <0.001 | <0.001 | 0.0025 | <0.001 | - | <0.001 | <0.001 | <0.001 | | <0.001 | <0.001 | <0.001 | <0.001 |  |
|  |  | Year*Class |  | - | - | - | - | - | - | - | - | - | | - | - | - | - |  |
|  |  | Season*Class |  | - | - | - | - | - | - | 0.035 | - | - | | - | - | - | - |  |

DM = dry matter; ME = metabolizable energy; CP = crude protein; EE = ether extract or crude fat; NfE = nitrogen-free extract or digestible carbohydrates.; TDF = total dietary fiber; COY = cubs of the year; YL = yearlings. *The sum of nutrients may exceed 100 % DM because of the protein-rich animal fiber that is also captured in the crude protein fraction of vertebrates and invertebrates, hence rendering values above 100. The sum of nutrients below 100 % are due to the ‘Miscellaneous’ fraction which was not attributed any nutrient content. The spring season was not included in statistical analyses and statistical output refers only to the comparison of the seasons summer and fall.

FIGURES


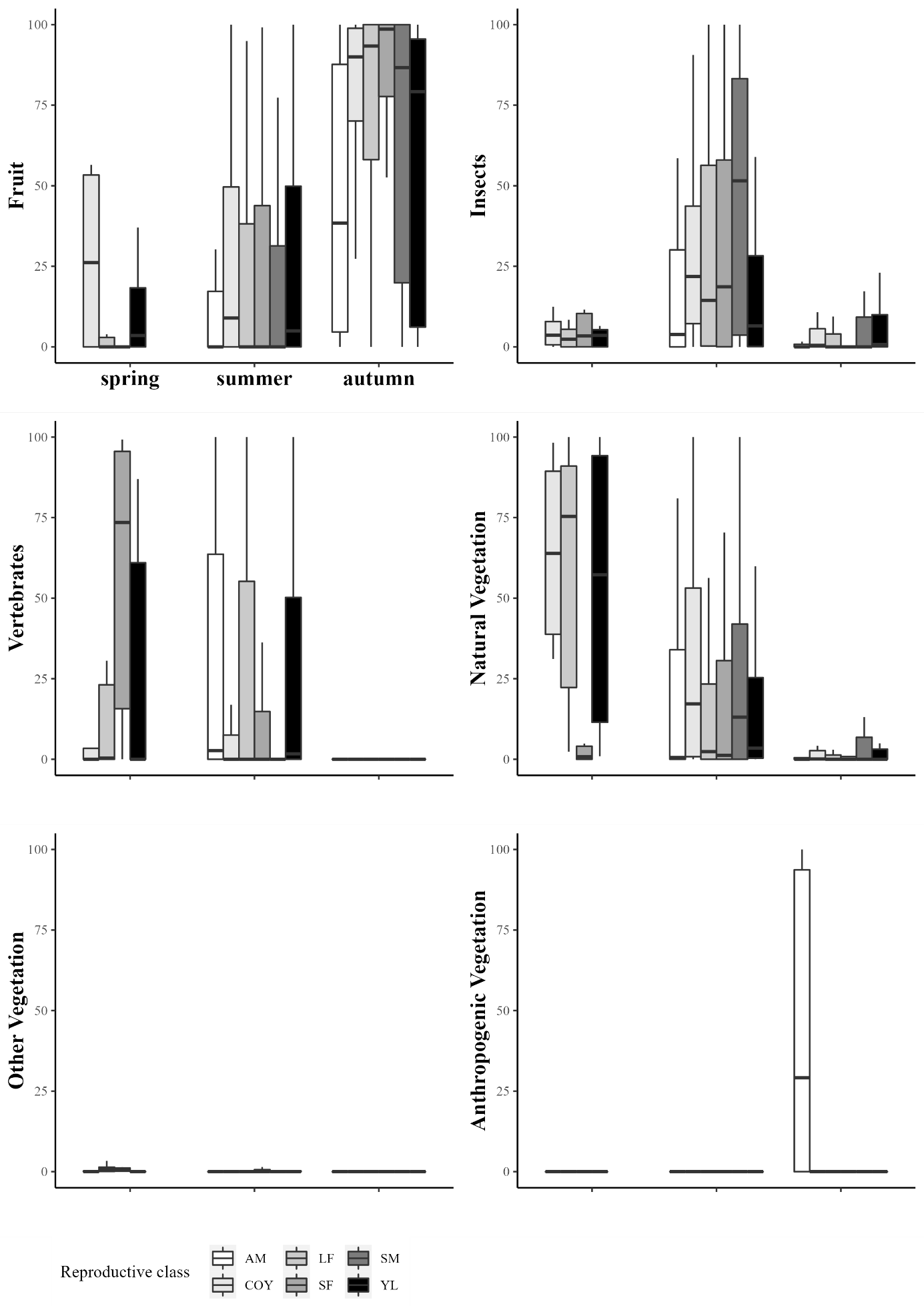


**Fig. A1 Average percentage of estimated dietary content (EDC) of ingredients per season and reproductive class in free-ranging Swedish brown bears.** AM = adult males; COY= females with cubs of the year; LF = adult lone females; SF = subadult females; SM = subadult males; YL = females with yearlings. The spring season was not included in statistical analyses and statistical output refers only to the comparison of the seasons summer and fall.


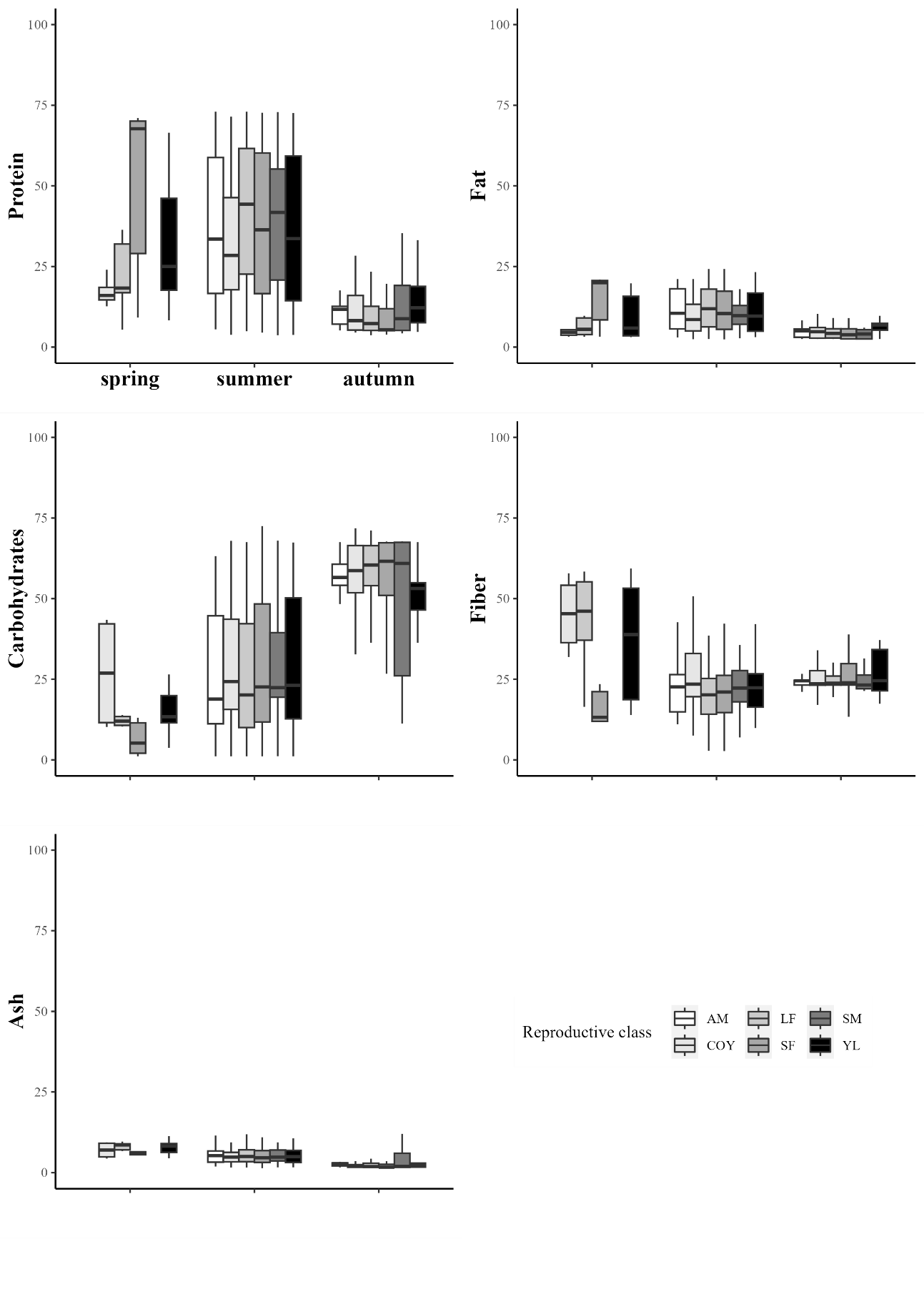


**Fig. A2 Average nutrient composition and ratios (% DM) of brown bear diets per season and reproductive class** DM = dry matter; AM = adult males; COY= females with cubs of the year; LF = adult lone females; SF = subadult females; SM = subadult males; YL = females with yearlings. The spring season was not included in statistical analyses and statistical output refers only to the comparison of the seasons summer and fall.


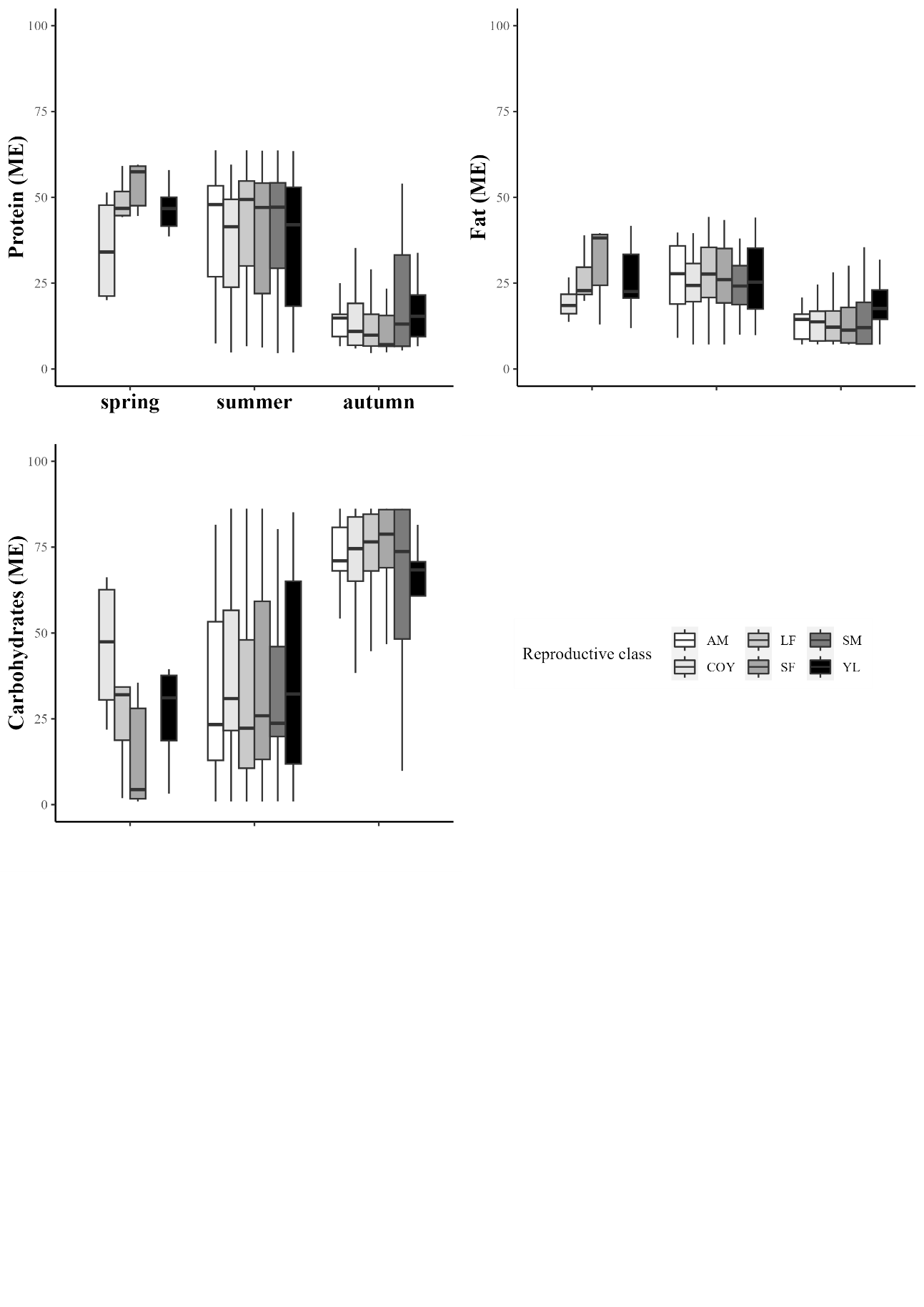


**Fig. A3 Average nutrient composition and ratios (% ME) of brown bear diets per season and reproductive class** ME = metabolizable energy; AM = adult males; COY= females with cubs of the year; LF = adult lone females; SF = subadult females; SM = subadult males; YL = females with yearlings. The spring season was not included in statistical analyses and statistical output refers only to the comparison of the seasons summer and fall.
