# Supplementary material for: Nutrient intake and its possible drivers in free‐ranging European brown bears (Ursus arctos arctos)
Source: Ecol Evol. 2023 May 30;13(5):e10156. doi: 10.1002/ece3.10156 (PMC10227639; doi:10.1002/ece3.10156)
Supplement: Supplementary file 2 — Appendix S2. [file ECE3-13-e10156-s002.docx]

**Nutrient intake and its possible drivers in free-ranging European brown bears (*Ursus arctos arctos*)**

Annelies De Cuyper^1^, Diederik Strubbe^2^, Marcus Clauss^3^, Luc Lens^2^, Andreas Zedrosser^4,5^, Sam Steyaert^6^, Leen Verbist^7^, Geert P. J. Janssens^1^

*^1^Department of Veterinary and Biosciences, Faculty of Veterinary Medicine, Ghent University, Merelbeke, Belgium*

*^2^Terrestrial Ecology Unit, Department of Biology, Faculty of Sciences, Ghent University, Ghent, Belgium*

*^3^Clinic for Zoo Animals, Exotic Pets and Wildlife, Vetsuisse Faculty, University of Zurich, Zurich, Switzerland*

*^4^Department of Natural Sciences and Environmental Health, Faculty of Technology, Natural Sciences and Maritime Sciences, University of South-Eastern Norway, Bø, Norway*

*^5^Institute for Wildlife Biology and Game Management, University for Natural Resources and Life Sciences, Vienna, Austria*

*^6^Faculty of Biosciences and Aquaculture, Nord University, Steinkjer, Norway*

*^7^Onderzoekskern Salto, Odisee Hogeschool, Campus Sint-Niklaas, Sint-Niklaas, Belgium*

**Appendix 2**

**Calculation of the theoretical minimal threshold non-fat to fat ratio ((CP+NfE):EE)**

We started from the basic reaction that 1 mol acetyl CoA reacts with 1 mol oxaloacetate to initiate the citric acid cycle and that carbohydrates, protein and fat can all render acetyl CoA and oxaloacetate (Mayes and Bender 2003, Akram 2014), except for fatty acids, which can only be converted to acetyl CoA. Based on this reaction we made the following calculations:

1. Carbohydrate pathway:

1 mol glucose 🡺 2 mol acetyl CoA or oxaloacetate (Kohlmeier 2015)

Molar mass glucose: 180.156 g/mol

90.078 g of glucose necessary for 1 mol of acetyl CoA or oxaloacetate

1. Fatty acid pathway:

The acetyl CoA yield from fatty acids depends on the fatty acid carbon chain length: after activation to fatty acyl CoA, the latter are mainly oxidized in the β-oxidation: 1 cycle of oxidation always splits off 2 C under the form of acetyl CoA (Bhagavan 2002, Kohlmeier 2015). If we use oleic acid (C18) as a representative fatty acid for brown bears (Käkelä and Hyvärinen 1996, Vranković et al. 2017), then we make the following calculation:

1 mol oleic acid 🡺 9 mol acetyl CoA

Molar mass oleic acid: 282.47 g/mol

31.39 g of oleic acid necessary for 1 mol of acetyl CoA

1. Protein pathway:

In most cases, 1 mol of amino acid leads to 1 mol of acetyl CoA (Kohlmeier 2015). If we use isoleucine as a representative amino acid (which has a molar mass close to the average of all amino acids), then we make the following calculation:

1 mol isoleucine 🡺 1 mol acetyl CoA or oxaloacetate

Molar mass isoleucine: 131.17 g/mol

131.17 g isoleucine necessary for 1 mol of acetyl CoA or oxaloacetate

Knowing that oxaloacetate cannot be produced from fatty acids, 1 mol acetyl CoA production from a fat source requires for instance 31.39 g oleic acid. One mol of oxaloacetate should be produced from 90.078 g of glucose or 131.17 g of isoleucine. To allow combustion of fatty acids in the citric acid cycle, the minimum glucose:fat ratio should then be 2.9 (g/g) in case carbohydrates would be the only glucogenic source. In case protein would be the only glucogenic source, the protein:fat ratio should minimally be 4.2 (g/g). When assuming an equal contribution of carbohydrates and protein to the citric acid cycle, the sum of carbohydrates and protein (in grams) should be at least 3.55 times the amount of fat to ensure that the latter can be combusted in the citric acid cycle. To express this ratio on a ME basis we multiply 3.55 with the unmodified Atwater factors which are factors for nutrients used in predictive equations for diet ME calculations of dogs and cats, and reflect the combustion heat, the digestibility and corrections for energy loss for protein in urine (16.72 kJ/g protein; 37.62 kJ/g fat; 16.72 kJ/g NfE) (NRC 2006): 3.55 x (16.72/37.62) = 1.58.

Bears will be able to deviate from this ratio if a substantial amount of dietary fat is not used for energy but for instance for storage (lipogenesis), hence by-passing the citric acid cycle. Bears can also have a lower ratio when only few oxaloacetate is used as substrate for other compounds than citrate, such as in gluconeogenesis, the urea cycle, the glyoxylate cycle, amino acid synthesis and fatty acid synthesis (<https://healthjade.net/oxaloacetate/>).

During hibernation, bears rely on their body fat reserves for energy supply (Nelson et al. 1983, Farley and Robbins 1995, Carey et al. 2003), hence assuming fat always needs a glucogenic resource for combustion seems counter-intuitive. Hibernation is marked by a great reduction in metabolism (Carey et al. 2003, Andrews 2007, Heldmaier 2011) together with a switch towards lipolytic metabolism and reduction of ‘carbolytic’ metabolism. The latter refers to a downregulation of the conversion of pyruvate (coming from carbohydrates or protein) towards acetyl CoA (Carey et al. 2003, Staples and Brown 2008). This means that acetyl CoA can still be produced from fatty acids (Mayes and Bender 2003, McDonald et al. 2011), and that the pathway of pyruvate conversion towards oxaloacetate (Mayes and Bender 2003) can still ensure citric acid functioning, even if at lower rates. The glycerol that is released from triglyceride breakdown typically serves as gluconeogenic substrate during hibernation (Carey et al. 2003), hence elevating oxaloacetate from cataplerosis, which makes a ‘lipogenic limitation’ irrelevant during such periods.

The minimum threshold value (1.58 on ME basis) of the non-fat to fat ratio should allow us to evaluate efficient metabolism independent of species: 1.5 for pine martens (Remonti et al. 2016), 1.7 for stone martens (Gazzola and Balestrieri 2020), 1.2 for wolves and feral cats (Plantinga et al. 2011, Bosch et al. 2015), 1.8 for domestic cats (Hewson-Hughes et al. 2011), 0.59 for domestic dogs (Hewson-Hughes et al. 2013). Given that for some species the ratio falls below the minimum threshold, implies that certain species may not be affected by this ratio for foraging decisions or that, as discussed above, only few oxaloacetate is used as substrate for other compounds than citrate, such as in gluconeogenesis, the urea cycle, the glyoxylate cycle, amino acid synthesis and fatty acid synthesis (<https://healthjade.net/oxaloacetate/>). In these studies, carbohydrate determination of diet items was done with different methods (NfE formula vs. non-structural carbohydrate calculation) which may cause variation in nutrient ratios.

References

Akram, M. 2014. Citric acid cycle and role of its intermediates in metabolism. - Cell Biochem. Biophys. 68: 475–478.

Andrews, M. T. 2007. Advances in molecular biology of hibernation in mammals. - BioEssays 29: 431–440.

Bhagavan, N. 2002. Lipids I: Fatty Acids and Eicosanoids. - In: Bhagavan, N. (ed), Medical Biochemistry. pp. 365–399.

Bosch, G. et al. 2015. Dietary nutrient profiles of wild wolves: insights for optimal dog nutrition? - Br. J. Nutr. 113: S40–S54.

Carey, H. V. et al. 2003. Mammalian hibernation: Cellular and molecular responses to depressed metabolism and low temperature. - Physiol. Rev. 83: 1153–1181.

Farley, S. D. and Robbins, C. 1995. Lactation, hibernation, and mass dynamics of American black bears and grizzly bears. - Can. J. Zool. 73: 2216–2222.

Gazzola, A. and Balestrieri, A. 2020. Nutritional ecology provides insights into competitive interactions between closely related Martes species. - Mamm. Rev. 50: 82–90.

Heldmaier, G. 2011. Life on low flame in hibernation. - Science (80-. ). 331: 866–867.

Hewson-Hughes, A. K. et al. 2011. Geometric analysis of macronutrient selection in the adult domestic cat, felis catus. - J. Exp. Biol. 214: 1039–1041.

Hewson-Hughes, A. K. et al. 2013. Geometric analysis of macronutrient selection in breeds of the domestic dog, Canis lupus familiaris. - Behav. Ecol. 24: 293–304.

Käkelä, R. and Hyvärinen, H. 1996. Site-specific fatty acid composition in adipose tissues of several Northern aquatic and terrestrial mammals. - Comp. Biochem. Physiol. - B Biochem. Mol. Biol. 115: 501–514.

Kohlmeier, M. 2015. Acetate. - In: Kohlmeier, M. (ed), Nutrient Metabolism: Structures, functions, and genes. pp. 147–153.

Mayes, P. A. and Bender, D. A. 2003. The citric acid cycle: The catabolism of acetyl-CoA. - In: Murray, R. K. et al. (eds), Harper’s Illustrated Biochemistry. pp. 130–135.

McDonald, P. et al. 2011. Animal Nutrition. - Pearson education.

Nelson, R. A. et al. 1983. Behavior, Biochemistry, and hibernation in black, grizzly, and polar bears. - Int. Conf. Bear Res. Manag. 5: 284–290.

NRC 2006. Nutrient requirements of dogs and cats. - National Academy Press.

Plantinga, E. A. et al. 2011. Estimation of the dietary nutrient profile of free-roaming feral cats: possible implications for nutrition of domestic cats. - Br. J. Nutr. 106: S35–S48.

Remonti, L. et al. 2016. Functional implications of omnivory for dietary nutrient balance. - Oikos 125: 1233–1240.

Staples, J. F. and Brown, J. C. L. 2008. Mitochondrial metabolism in hibernation and daily torpor: A review. - J. Comp. Physiol. B Biochem. Syst. Environ. Physiol. 178: 811–827.

Vranković, L. et al. 2017. The lipid composition of subcutaneous adipose tissue of brown bears (Ursus arctos) in Croatia. - Physiol. Biochem. Zool. 90: 399–406.
